# Revision of the genus *Furusawaia* Chûjô, 1962 (Coleoptera, Chrysomelidae, Galerucinae)

**DOI:** 10.3897/zookeys.1057.71451

**Published:** 2021-08-27

**Authors:** Chi-Feng Lee, Jan Bezděk

**Affiliations:** 1 Applied Zoology Division, Taiwan Agricultural Research Institute, Taichung 413, Taiwan Taiwan Agricultural Research Institute Taichung Taiwan; 2 Mendel University in Brno, Department of Zoology, Fisheries, Hydrobiology and Apiculture, Zemĕdĕlská 1, 613 00 Brno, Czech Republic Mendel University in Brno Brno Czech Republic

**Keywords:** Caryophyllaceae, citizen scientists, Food plant, leaf beetles, new species, new synonym, *
Stellaria
*, taxonomy, winglessness, *
Yunnaniata
*

## Abstract

*Yunnaniata* Lopatin, 2009 is regarded as a junior synonym of *Furusawaia* Chûjô, 1962 **syn. nov**. *Yunnaniatakonstantinovi* Lopatin, 2009 **comb. nov.** is transferred to the genus *Furusawaia* Chûjô and redescribed. *Furusawaiacontinentalis* Lopatin, 2008 and *F.yosonis* Chûjô are recognized as valid species and redescribed. Four new species are described from Taiwan: *F.jungchani***sp. nov.**, *F.lui***sp. nov.**, *F.tahsiangi***sp. nov.**, and *F.tsoui***sp. nov.** A key to Taiwanese and Chinese species of *Furusawaia* is provided.

## Introduction

*Furusawaia* Chûjô, 1962 is a little known galerucine genus, with *F.yosonis* Chûjô from Taiwan as the type and only species. No additional species were described until Lopatin (2008) described the second species, *F.continentalis* Lopatin from China (Yunnan, Sichuan). Furusawaia Chûjô was initially placed in the section Hylaspites within the tribe Sermylini (Wilcox 1971, 1975). It was transferred to section Capulites by Seeno and Wilcox (1982), containing four genera: *Capula* Jacobson, 1925; *Furusawaia* Chûjô, 1962; *Nepalogaleruca* Kimoto, 1970; and Himaplosonyx Chen, 1976. The section Capulites was redefined byBezdĕk and Beenen (2009) and *Yunnaniata* Lopatin, 2009 (in Lopatin and Konstantinov 2009) was included and considered closely related to *Furusawaia* Chûjô. The taxonomic status of both genera was re-evaluated in the present study.

The Taiwan Chrysomelid Research Team (TCRT) was founded in 2005 and is composed of ten members. All of them are amateurs interested in producing a complete inventory of chrysomelid species in Taiwan. Specimens of *Furusawaia* are difficult to collect, with only a few individuals collected on or under stones along forest trails at mid-altitudes (above 2,000 m). This habitat is similar to that of *Yunnaniatakonstantinovi* (Bezdĕk and Beenen 2009). Due to this problem, more citizen scientists were recruited using internet social media. Pin-Hsun Ko (柯品薰) was the first person to observe that adults of *Furusawaia* species fed on leaves of *Stellariamedia* (L.) Vill (Caryophyllaceae) on March 30, 2015. Members of the TCRT started to focus searches for adults where these food plants grew. However, specimens were still difficult to find during numerous field trips, with one exception. Ten individuals were collected from Huakang (華崗) by Jung-Chan Chen (陳榮章) on April 24, 2019 (see *F.jungchani* sp. nov.). Fortunately, many more were collected thanks to the efforts of citizen scientists resulting in 96 specimens that were available for this study.

## Materials and methods

For taxonomic study, the abdomens of adults were separated from the forebodies and boiled in 10% KOH solution, followed by washing in distilled water to prepare genitalia for illustrations. The genitalia were then dissected from the abdomens, mounted on slides in glycerin, and studied and drawn using a Leica M165 stereomicroscope. A Nikon ECLIPSE 50i microscope was used for detailed examinations.

At least two pairs from each species were examined to delimit variability of diagnostic characters. For species collected from more than one locality, at least one pair from each locality was examined. Length was measured from the anterior margin of the eye to the elytral apex, and width at the greatest width of the elytra.

Specimens studied herein are deposited at the following institutes and collections:

**BPBM**Bernice P. Bishop Museum, Hawaii, USA [James Boone];

**IZAS** Institute of Zoology, Chinese Academy of Sciences, Beijing, China [Ruie Nie];

**JBCB** Jan Bezděk collection, Brno, Czech Republic;

**KMNH**Kitakyushu Museum of Natural History and Human History, Kitakyushu, Japan [Yûsuke Minoshima];

**TARI**Taiwan Agricultural Research Institute, Taichung, Taiwan;

**USNM**Smithsonian Institution, National Museum of Natural History, Washington, U.S.A. [Alexander S. Konstantinov];

**TCRT** Taiwan Chrysomelid Research Team;

**ZIN**Zoological Institute, Russian Academy of Sciences, St. Peterburg, Russia [Alexey Moseyko];

Exact label data are cited for all type specimens of previously described species; a double slash (//) divides the data on different labels and a single slash (/) divides the data in different rows. Other comments and remarks are in square brackets: [p] – preceding data are printed, [h] – preceding data are handwritten, [w] – white label, [r] – red label, [b] – blue label.

**Figure 1.**
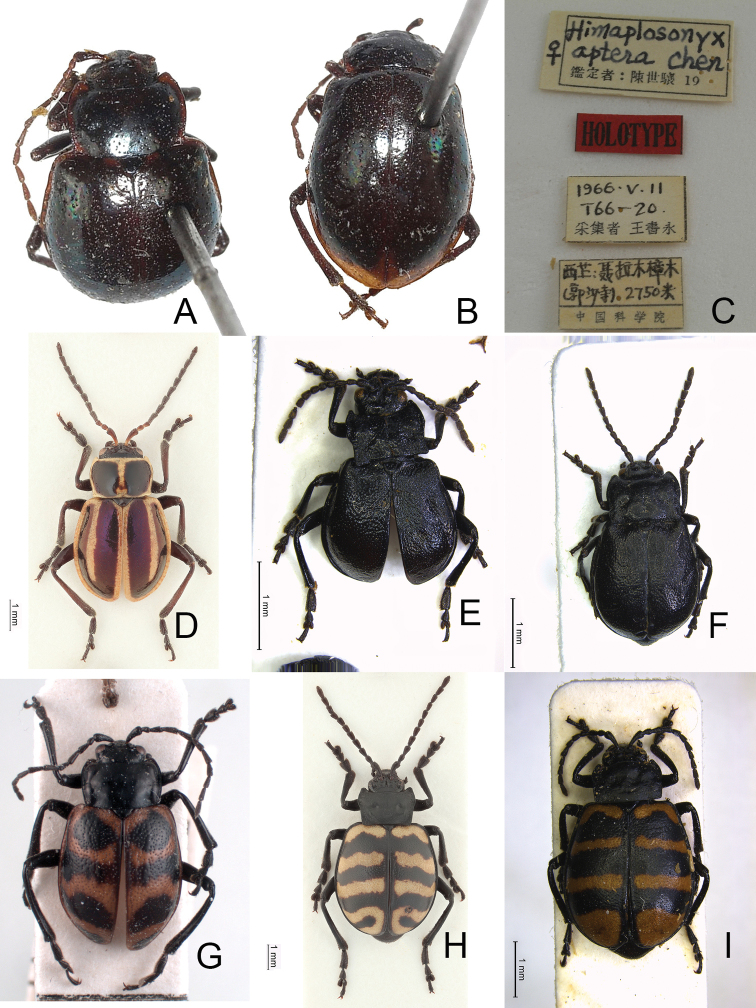
Habitus, dorsal view **A***Himaplosonyxapterus* Chen, holotype, female, front view **B** ditto, back view **C** same, labels **D***Neplogalerucaangustilineata* Kimoto & Takizawa, male **E***Capulaapicalis* Chen et al., male **F***C.caudata* Chen et al., female **G***Furusawaiacontinentalis* Lopatin, holotype, male **H***F.konstantinovi* (Lopatin), male **I** same species, female.

Identified specimens of the following species are included in the study:

*Himaplosonyxapterus* Chen, 1976 (Fig. 1A–C): holotype ♀ (IZAS, based on photographs): “Himaplosonyx / ♀ aptera Chen [h] / 鑑定者 [identifier] : 陳世驤 [Sicien Chen] 19 [p, w] // HOLOTYPE [p, r] // 1966.V.11 / T66-20 [p] / 采集者 [collector] 王書永 [Shu-Yung Wang] [p, w] // 西藏 [Tibet]: 聂拉木 [Nielamu] 樟木 [Camphor wood] / (郭沙寺[Guosha Temple]) 2750米 [2750 m] / 中國科學院 [Chinese Academy of Sciences] [p, w]”.

*Nepalogalerucaangustilineata* Kimoto & Takizawa, 1972 (Fig. 1D): NEPAL. 1♂ (JBCB), Chomrong, 9.V.1994, leg. P. Hesoun.

*Nepalogalerucaelegans* Kimoto, 1970: Nepal. 1♂ (KMNH), Prov. Nr. 3 East Dudh Kosi Tal under Thangpoche, 3400 m, 29–31.V.1964, leg. W. Dierl; 1♀ (KMNH), Prov. Nr. 3 East Jubing, 1600 m, 2.V. 1964, leg. W. Dierl; 1♂ (KMNH), C. Baroni U., Thodung via Those, 3100 m, 29–31.V.1976, leg. W. Wittmer.

*Capulaapicalis*Chen et al., 1986 (Fig. 1E): China. Sichuan: 1♂ (JBCB), Sa’de env., alpine meadows, 29°36.4'N, 101°22.9'E, 4500 m, V.2004, leg. Hackel & Sehnal.

*Capulacaudata*Chen et al., 1986 (Fig. 1F): China. Sichuan: 1♀ (JBCB), Sabde, 4200 m, 29°04'168"N, 101°25'720"E, 25.V.2001, leg. M. Janata.

## Taxonomic results

### Furusawaia

Taxon classificationAnimaliaColeopteraChrysomelidae

Chûjô, 1962

CA91619B-9282-5E7C-8137-568012F311B1

Furusawaia Chûjô, 1962: 107 (type species: Furusawaiayosonis Chûjô, 1962, by original designation); Wilcox 1971: 210 (catalogue); Kimoto and Chu 1996: 91 (catalogue); Kimoto and Takizawa 1997: 298 (key); Beenen 2010: 458 (catalogue); Yang et al. 2015: 187 (catalogue).
Yunnaniata Lopatin in Lopatin and Konstantinov 2009: 8 (type species: Yunnaniatakonstantinovi Lopatin, 2009, by original designation); Yang et al., 2015: 184 (catalogue). syn. nov.

#### Included species.

*Furusawaiacontinentalis* Lopatin, 2008, *F.konstantinovi* (Lopatin, 2009) comb. nov., *F.jungchani* sp. nov., *F.lui* sp. nov., *F.tahsiangi* sp. nov., *F.tsoui* sp. nov., and *F.yosonis* Chûjô, 1962.

#### Diagnosis.

**Figure 2.**
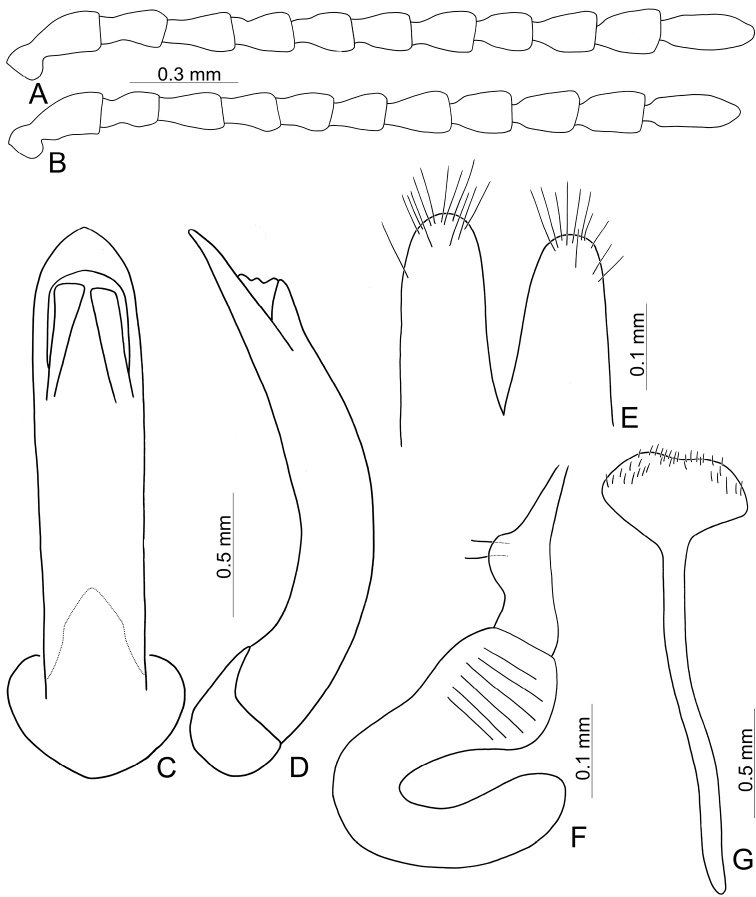
Diagnostic characters of *Capulaapicalis* Chen et al. (**A, C, D**) and *C.caudata* Chen et al. (**B, E–G**) **A** antenna, male **B** antenna, female **C** aedeagus, dorsal view **D** ditto, lateral view **E** gonocoxae **F** Spermatheca **G** abdominal ventrite VIII, female.

Adults of *Furusawaia* Chûjô are similar to those of *Capula* Jacobson in possessing a black general color pattern and midcoxae widely separated, the distance between them at least as wide as half of transverse diameter of coxa; but they differ from those of *Capula* Jacobson by the red or orange transverse stripes on the elytra (elytra entirely metallic or black in *Capula* (Fig. 1E, F), humeral calli absent (humeral calli present in *Capula*), antenna elongate, length to width ratios of antennomeres IV-VII more than 1.9 × (antennae shorter, length to width rations of antennomeres IV–VII 1.5–1.7 ×, length to width ratios of antennomeres I–XI in male of *Capulaapicalis* 2.4: 1.9: 1.8: 1.6: 1.5: 1.5: 1.6: 1.6: 1.7: 1.5: 2.7 (Fig. 2A); length to width ratios of antennomeres I–XI in female of *Capulacaudata* 2.3: 1.7: 1.8: 1.5: 1.5: 1.5: 1.6: 1.5: 1.6: 1.6: 3.0 (Fig. 2B)). Genitalic characters are also diagnostic. In *Capula*, the aedeagus lacks endophallic sclerites (Fig. 2C, D) (at least primary endophallic sclerite present in *Furusawaia*), the gonocoxae (Fig. 2E) of *Capula* females are much wider than those of *Furusawaia* and the spermatheca (Fig. 2F) possesses a short and strongly swollen receptacle (elongate and slightly swollen receptacle of spermatheca in *Furusawaia*). Abdominal ventrites VIII (Fig. 2G) are similar in both genera. Members of *Himaplosonyx* (Fig. 1A, B) differ from those of *Furusawaia* in possessing explanate lateral margins of the pronotum, disc with two foveae in the middle, and the pronotal anterior margin bordered but posterior marginal border absent. Members of *Nepalogaleruca* (Fig. 1D) differ from those of *Furusawaia* in the yellow general color pattern, pronotum with two longitudinal black or metallic spots, elytra with two pairs of longitudinal black or metallic stripes, and the midcoxae closer to each other, distance between them less than half of transverse diameter of coxa.

#### Remarks.

Bezdĕk and Beenen (2009) separated *Yunnaniata* from *Furusawaia* based on the flat pronotum, however, convexity of the pronotum varies among different species and populations of *Furusawaia* in Taiwan. Thus, both genera are regarded as synonyms here. Although *Yunnaniata* males possess more complicated sclerites in the aedeagus, no additional characters separate the two genera.

#### Biology.

**Figure 3.**
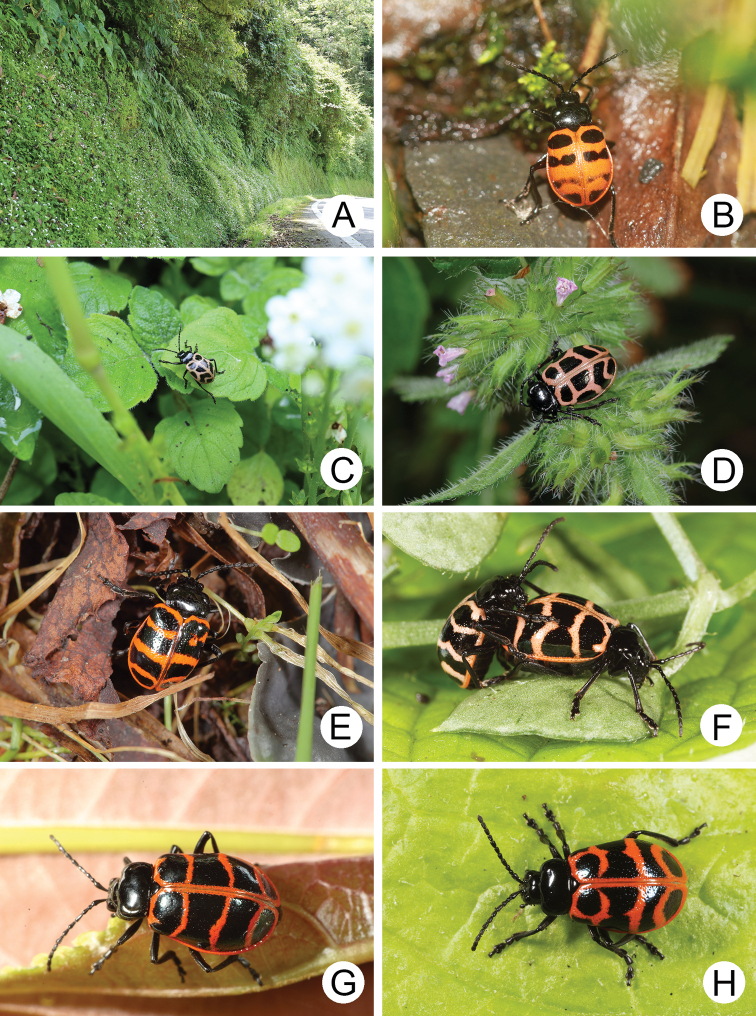
Field photographs of *Furusawaia* species **A** microhabitat for *F.lui* sp. nov. in Hsinpaiyang (新白楊) **B** adult of *F.jungchani* sp. nov. in the daytime, Huakang (華崗) **C** adult of *F.lui* sp. nov. in the daytime, Hsinpaiyang (新白楊) **D** adult of *F.lui* sp. nov. at night, Hsinpaiyang (新白楊) **E** adult of *F.tahsiangi* sp. nov. in the daytime, Hsuehshan (雪山) **F** adult of *F.tsoui* sp. nov. at night, Jianqing trail (見晴步道) **G** adult of *F.yosonis* at night, Alishan (阿里山) **H** adult of an undescribed species, Tianchi Lodge (天池山莊)

Adults were observed walking or resting on forest trails (e.g., Fig. 3A) at low altitudes (above 1,000 m) in northern Taiwan or middle and high altitudes (above 2,000 m) in China, central and southern Taiwan. They feed on leaves of *Stellaria* species (Caryophyllaceae) in Taiwan.

#### Distribution.

West China (Yunnan, Sichuan), Taiwan.

### Chinese species

#### Furusawaia continentalis

Taxon classificationAnimaliaColeopteraChrysomelidae

Lopatin, 2008

87973321-598B-5980-A9ED-0403FFF2D873

Figs 1G , 4A, D, E

Furusawaia continentalis Lopatin, 2008: 925; Beenen 2010: 458 (catalogue).

##### Types.

***Holotype*** ♂ (ZIN): “**CH**. Yunnan. N. Baoshan / 25 29 10 N / 99 04 38 E / H 3530 m, 10.05 2006 / Belousov & Kabak leg [p, w] // Holotypus [p, r] // Furosawaia [sic!] / continentalis sp. n. [h] / det. I. Loptatin, 200[p]8[h, w]”.

##### Other material.

China. Yunnan: 1♂ (JBCB), 30 km mer.-occ ad Daochang, 2800 m, 9.VI.2001, leg. local collector.

##### Redescription.

**Figure 4.**
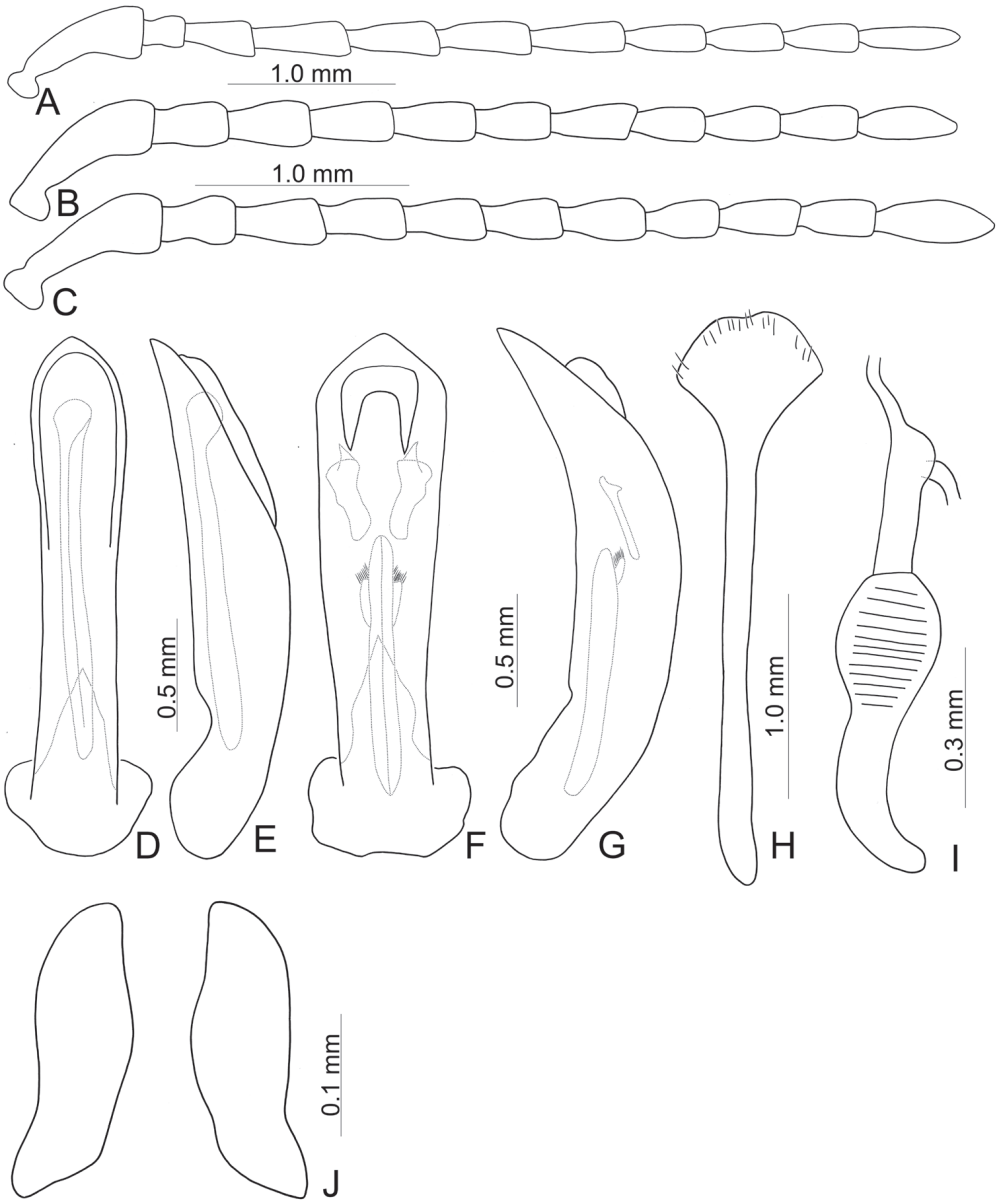
Diagnostic characters of *Furusawaiacontinentalis* Lopatin and (**A, D, E**) and *Furusawaiakonstantinovi* (Lopatin) comb. nov. (**B, C, F–J**) **A, B** antenna, male **C** antenna, female **D, F** aedeagus, dorsal view **E, G** ditto, lateral view **H** abdominal ventrite VIII, female **I** spermatheca **J** gonocoxae.

**Male**: Length 8.1 mm, width 4.5 mm. Body color (Fig. 1G) black, elytra with red circular stripes along basal and lateral margins, anterior transverse red stripe at basal 1/4, red stripe along basal margin extending downwards at suture and humeral calli connected with anterior red stripe, two oblique stripes at middle and apical 1/4. Antennae filiform in males (Fig. 4A), length ratios of antennomeres I–XI 1.0: 0.3: 0.5: 0.7: 0.6: 0.6: 0.6: 0.6: 0.5: 0.5: 0.7, length to width ratios of antennomeres I–XI 2.8: 1.4: 1.9: 2.8: 2.7: 2.9: 3.0: 3.0: 2.7: 2.6: 4.4. Pronotum 1.8 × wider than long, disc generally flat; dull, with reticulate microsculpture; with sparse minute punctures confused with few fine, but slightly larger, punctures, with lateral groove extending posterior to base; basal margin straight; apical margin moderately concave; anterior angles obtuse; lateral margins distinct and rounded. Elytra with rounded lateral margins, widest behind middle, 1.2 × longer than wide; disc with dense, coarse punctures; shining, without reticulate microsculpture. Aedeagus (Fig. 4D, E) slender in dorsal view, 7.2 × longer than wide, parallel-sided, strongly narrowed apically, apex narrowly rounded; ostium large, covered by membrane; slightly curved in lateral view; endophallic sclerite extremely elongate, 0.7 × as long as aedeagus.

##### Diagnosis.

Adults of *Furusawaiacontinentalis* can be recognized by the following combination of characters: elongate antennae, length to width ratios of antennomeres IV–X more than 2.5 × (less than 2.5 × in others); disc of pronotum generally flat (more or less convex in Taiwanese species), dull and with reticulate microsculpture (only shared with *F.konstantinovi*), apical margin moderate concave (straight apical margin in others); anterior angles obtuse (anterior angles strongly produced to distinct bulb in others); disc of elytra smooth, lacking reticulate microsculpture, with dense coarse punctures (disc dull, with reticulate microsculpture and dense coarse punctures in *F.konstantinovi*; disc smooth, lacking reticulate microsculpture but with sparse punctures in Taiwanese species); red stripe along suture abbreviated behind anterior stripe at basal 1/3 (Fig. 1E) (red stripe entirely absent in *F.konstantinovi* (Fig. 1E, F), but present in Taiwanese species). In males of *F.continentalis*, aedeagus (Fig. 4D, E) with elongate primary endophallic sclerite, 0.7 × as long as aedeagus (small primary endophallic sclerite, 0.4–0.5 × as long as aedeagus), without lateral expansions near apex (with lateral expansions near apex in others).

##### Food plants.

Unknown.

##### Distribution.

China: Sichuan, Yunnan.

#### Furusawaia konstantinovi

Taxon classificationAnimaliaColeopteraChrysomelidae

(Lopatin, 2009) comb. nov.

70E17D28-7074-50C0-8043-98B6008923B4

Figs 1H, I , 4B, C, F–J

Yunnaniata konstantinovi Lopatin in Lopatin and Konstantinov 2009: 10; Bezdĕk and Beenen 2009: 46 (redescription); Yang et al. 2015: 184 (catalogue).

##### Types.

***Holotype*** ♂ (USNM, by monotype): “China. Yunnan, Lijiang 29. V. / Yulongshan, Prim. forest 2002 / 2800 m N27°08'20", E100°14'6" / leg. A. Konstantinov & M. Volkovitsh [p, w] // Yunnaniata / konstantinovi sp. n. [h] / det. I. Lopatin, 200[p]5[h, w] // Holotypus [p, r] // BLNO / 002622 [p, b] // USNMENT / 00871439 [p, w]”.

##### Other material.

China. Yunnan: 4♂ 1♀ (JBCB, 1♂ TARI), 32 km N. Lijiang, 21.VI.2007, Maoniuping (Yak meadows), 27°9.9'N, 100°14.5E, 3540 m, leg. J. Hájek & J. Růžička [Individually collected on wet vegetation and under stones and logs, wet yak pasture]; 1♀ (TARI); Yulong Mts., 4000 m, 27.V.1993, leg. Bolm.

##### Redescription.

Length 8.0–9.3 mm, width 4.8–5.2 mm. Body color (Fig. 1H, I) black, elytra four transverse yellow stripes behind base, at basal 1/4, middle, and apical 1/4 respectively, three anterior stripes straight, posterior stripe curved posteriorly near suture and extending into apex; in some individuals the entire apical 1/4 is yellow except the margin. Antennae filiform in males (Fig. 4B), length ratios of antennomeres I–XI 1.0: 0.4: 0.5: 0.5: 0.5: 0.4: 0.5: 0.4: 0.4: 0.5: 0.6, length to width ratios of antennomeres I–XI 3.1: 1.7: 1.7: 2.1: 2.1: 2.0: 2.2: 2.1: 2.1: 2.2: 3.0; similar in females (Fig. 4C), length ratios of antennomeres I–XI 1.0: 0.4: 0.5: 0.5: 0.5: 0.5: 0.5: 0.4: 0.5: 0.5: 0.7, length to width ratios of antennomeres I–XI 2.8: 1.6: 1.9: 2.0: 1.9: 1.9: 2.1: 2.0: 2.1: 2.1: 2.9. Pronotum 1.6–1.7 × wider than long, disc generally flat; dull, with reticulate microsculpture; with sparse fine confused punctures mixed with a few coarse punctures, with lateral shallow depressions; lateral margins distinct, more so in males, less so in females; apical and basal margins straight; anterior angles strongly produced to distinctly acute angles. Elytra with rounded lateral margin, widest behind middle, 1.1–1.2 × longer than wide; disc dull, with reticulate microsculpture, and dense, fine punctures. Aedeagus (Fig. 4F, G) slender in dorsal view, sides gradually narrowed to base, strongly, abruptly narrowed subapically, apex narrowly rounded; ostium with one median longitudinal sclerite; strongly curved in lateral view; primary endophallic sclerite elongate, 0.4 × as long as aedeagus, one pair of short lateral expansions near apex, covered with fine setae, and with one additional pair of short longitudinal sclerites above apex of primary endophallic sclerite, with irregular margins and apical horns. Gonocoxae (Fig. 4J) reduced into one pair of small flattened sclerites. Ventrite VIII (Fig. 4H) with apex well sclerotized and extremely small, several short setae along apical margin, spiculum extremely long. Receptacle of spermatheca (Fig. 4I) slightly swollen, separated from pump; pump short and slightly curved; sclerotized proximal spermathecal duct separated from receptacle, moderately long, with wide base.

##### Diagnosis.

Adults of *Furusawaiakonstantinovi* can be recognized by the following combination of characters: disc of pronotum generally flat (more or less convex in Taiwanese species), dull and with reticulate microsculpture (only shared with *F.continentalis*), lateral margin narrowed at posterior half (lateral margins rounded in others); disc of elytra dull, with reticulate microsculpture, with dense coarse punctures (disc smooth, lacking reticulate microsculpture and dense coarse punctures in *F.continentalis*; disc smooth but with sparse punctures in Taiwanese species); suture and lateral margins of elytra black (Fig. 1E, F) (lateral margins and at least part of suture of elytra with stripes in others). In males of *F.continentalis*, aedeagus (Fig. 4F, G) with one additional pair of small elongate endophallic sclerites above primary endophallic sclerite (no additional sclerites in others).

##### Food plants.

Unknown.

##### Distribution.

China: Yunnan.

### Taiwanese species

#### Furusawaia jungchani sp. nov.

Taxon classificationAnimaliaColeopteraChrysomelidae

BACB7CD4-625C-5B1B-B642-1AEE897C62B2

http://zoobank.org/FCD26A55-C260-4977-A894-B25BA485A9E7

Figs 3B , 5 , 6 , 7

##### Types (n = 19).

***Holotype*** ♂ (TARI), Taiwan. Nantou: Huakang (華崗), 10.IV.2019, leg. J.-C. Chen (陳榮章). ***Paratypes***. 3♂, 2♀ (TARI), same data as holotype; 8♂, 2♀ (TARI), same but with “24.IV.2019”; 1♂, 1♀ (TARI), same but with “23.V.2017”; 1♂ (TARI), Hohuansi trail (合歡溪步道 = Huakan, 華崗), 15.V.2017, leg. J.-C. Chen.

##### Description.

**Figure 5.**
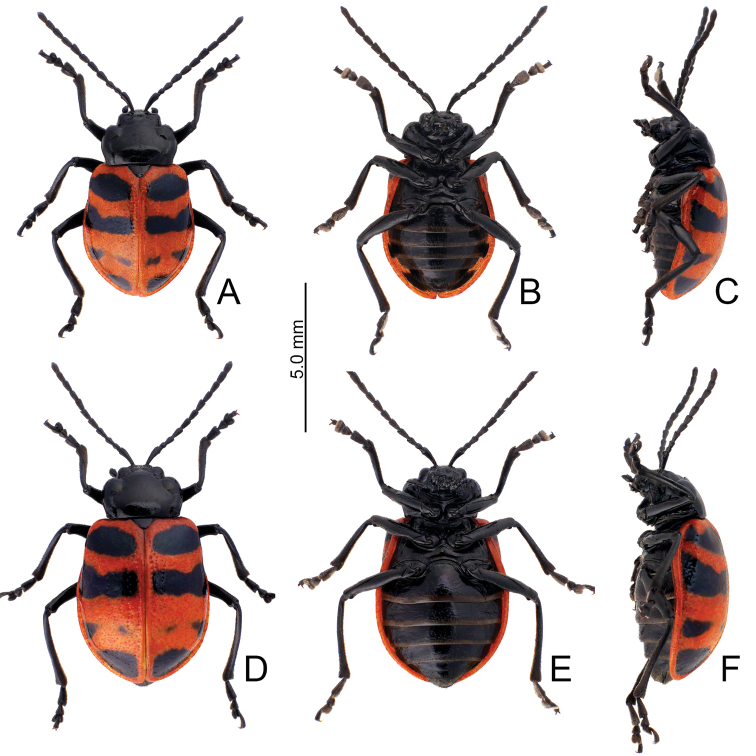
Habitus, *Furusawaiajungchani* sp. nov. **A** male, dorsal view **B** ditto, ventral view **C** ditto, lateral view **D** female, dorsal view **E** ditto, ventral view **F** ditto, lateral view.

**Figure 6.**
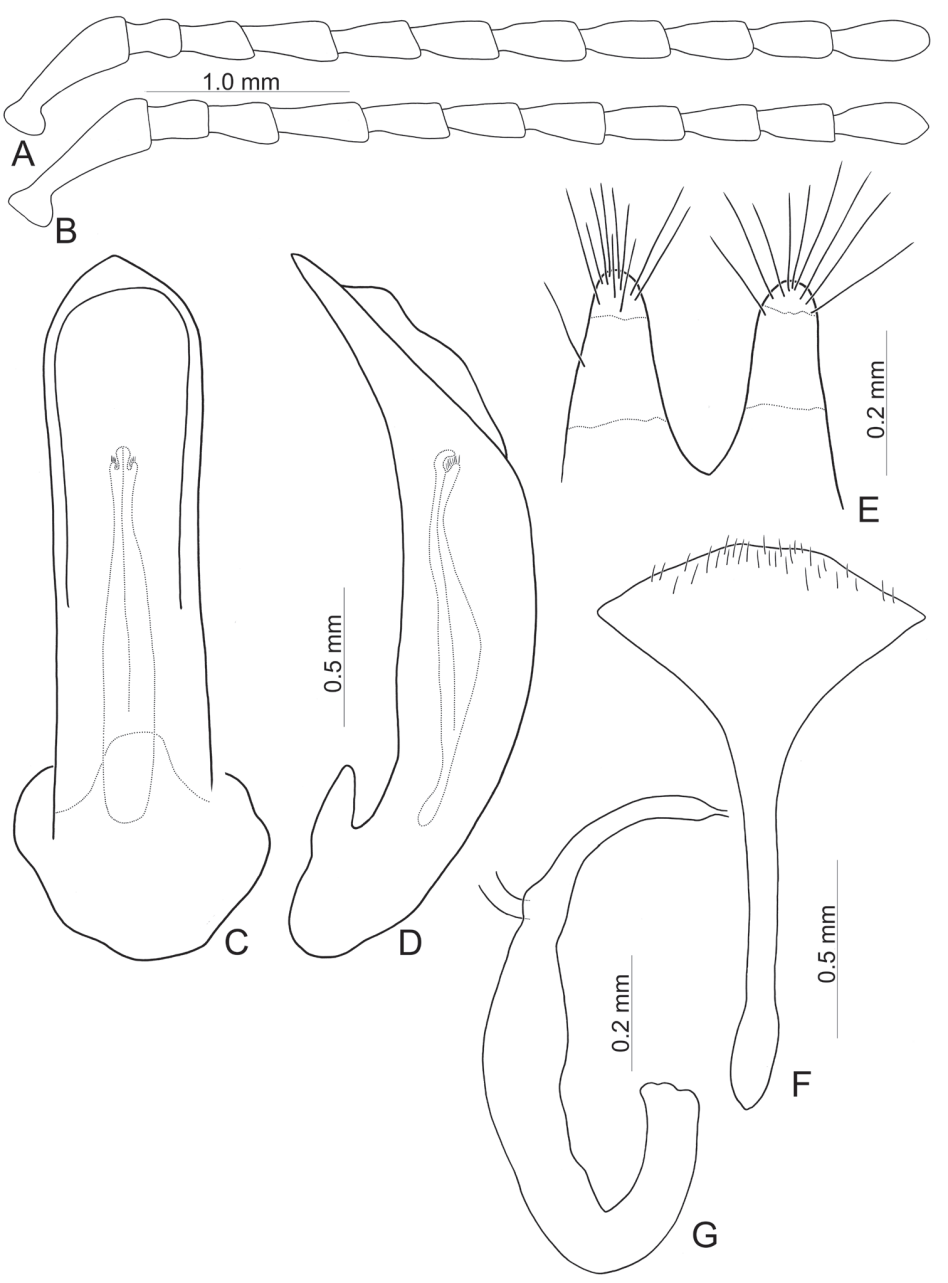
Diagnostic characters of *Furusawaiajungchani* sp. nov. **A** antenna, male **B** antenna, female **C** aedeagus, dorsal view **D** ditto, lateral view **E** gonocoxae **F** abdominal ventrite VIII, female **G** spermatheca.

Length 6.8–8.3 mm, width 4.1–5.1 mm. Body color (Fig. 5) black, elytra with red or orange stripes along basal and lateral margins, and suture, three transverse red or orange stripes at basal 1/4, middle, and apical 1/4 respectively, anterior and median stripes straight, posterior stripe curved anteriorly at middle, median and posterior stripes wider, sometimes connected with each other, in some individuals anterior stripe also wider. Antennae filiform in males (Fig. 6A), length ratios of antennomeres I–XI 1.0: 0.3: 0.4: 0.6: 0.6: 0.5: 0.6: 0.6: 0.5: 0.5: 0.6, length to width ratios of antennomeres I–XI 3.2: 1.4: 1.7: 2.2: 2.3: 2.1: 2.3: 2.3: 2.1: 2.2: 2.4; similar in females (Fig. 6B), length ratios of antennomeres I–XI 1.0: 0.4: 0.4: 0.5: 0.5: 0.5: 0.5: 0.5: 0.5: 0.5: 0.6, length to width ratios of antennomeres I–XI 3.0: 1.5: 1.6: 2.2: 2.1: 2.1: 1.9: 2.4: 2.1: 2.0: 2.4. Pronotum 1.7–1.8 × wider than long, disc strongly convex; smooth, without reticulate microsculpture; with punctures obsolete, with lateral impressions; lateral margins distinct, rounded, and widest at apical 1/3, reduced at anterior angles; apical and basal margins straight; anterior angles strongly produced to a bulbous point. Elytra with rounded lateral margins, widest behind middle, 1.2–1.3 × longer than wide; disc smooth, without reticulate microsculpture; and with sparse, coarse punctures. Aedeagus (Fig. 6C, D) slender in dorsal view, 5.7 × longer than wide, parallel-sided, narrowed near apex, apex narrowly rounded; ostium large, covered by membrane; strongly curved in lateral view; endophallic sclerite elongate, 0.5 × as long as aedeagus, one pair of short lateral expansions near apex, covered with fine setae; basal 2/3 widened and parallel-sided. Only median areas of apices of gonocoxae (Fig. 6E) sclerotized, elongate, apex narrowly rounded, with several long setae near apex. Ventrite VIII (Fig. 6F) with apex well sclerotized and small, several short setae along apical margin, spiculum long. Receptacle of spermatheca (Fig. 6G) slightly swollen, undivided from pump; pump long and strongly curved; sclerotized proximal spermathecal duct undivided from receptacle, moderately long.

##### Diagnosis.

Adults of *Furusawaiajungchani* sp. nov. are similar to those of *F.tahsiangi* sp. nov. in sharing straight median and posterior stripes on the elytra but differ by the wider median and posterior stripes (Fig. 5) (median and posterior stripes not so modified in *F.tahsiangi* sp. nov. (Fig. 10)); and strongly convex pronotum with reduced lateral margin at anterior angles (less convex pronotum with lateral margin at anterior angles in *F.tahsiangi* sp. nov.). In males of *F.jungchani* sp. nov., the aedeagus is strongly curved in lateral view (Fig. 6D) (moderately curved in *F.yosonis* (Fig. 15D), slightly curved in others (Figs 9F, 11D, 13D)); endophallic sclerite (Fig. 6C) similar to that of *F.yosonis* (Fig. 15C) with basal 2/3 wider and parallel-sided (only wider at middle in *F.lui* sp. nov. (Fig. 9C); basal 2/3 widened but basally narrowed, and strongly widened at middle in *F.tahsiangi* sp. nov. (Fig. 11C); basal 2/3 widened but basally and in basal 3/7 narrowed in *F.tsoui* sp. nov. Fig. 13C)). In females of *F.jungchani* sp. nov., the spermathecae (Fig. 6G) are similar to those of *F.yosonis* (Fig. 15G) with slightly swollen receptacle (moderately swollen receptacle in *F.lui* sp. nov. (Fig. 9I) and *F.tahsiangi* sp. nov. (Fig. 11F); strongly swollen receptacle in *F.tsoui* sp. nov. (Fig. 13G)) and apex undivided from sclerotized proximal duct (Fig. 6G) (apex truncate and divided from sclerotized proximal duct in *F.lui* sp. nov. (Fig. 9G, I) and *F.tahsiangi* sp. nov. (Fig. 11F)); abdominal ventrite VIII (Fig. 6F) similar to that of *F.lui* sp. nov. (Fig. 9H) with large, well sclerotized apex (membranous apex in *F.tsoui* sp. nov. (Fig. 13F) and *F.yosonis* (Fig. 15F); sclerotized and small apex in *F.tahsiangi* sp. nov. (Fig. 11G)); gonocoxae (Fig. 6E) similar to those of *F.tahsiangi* sp. nov. (Fig. 11E) and *F.tsoui* sp. nov. (Fig. 13E) with rounded apices (acute apices in *F.yosonis* (Fig. 15E)) and dense setae present only at apical area (dense setae present at apical and lateral area in *F.lui* sp. nov. (Fig. 9J)).

##### Food plants.

*Stellariareticulivena* Hayata (Caryophyllaceae).

##### Biological notes.

All adults were found on forest trails during daytime (Fig. 3B).

##### Distribution.

**Figure 7.**
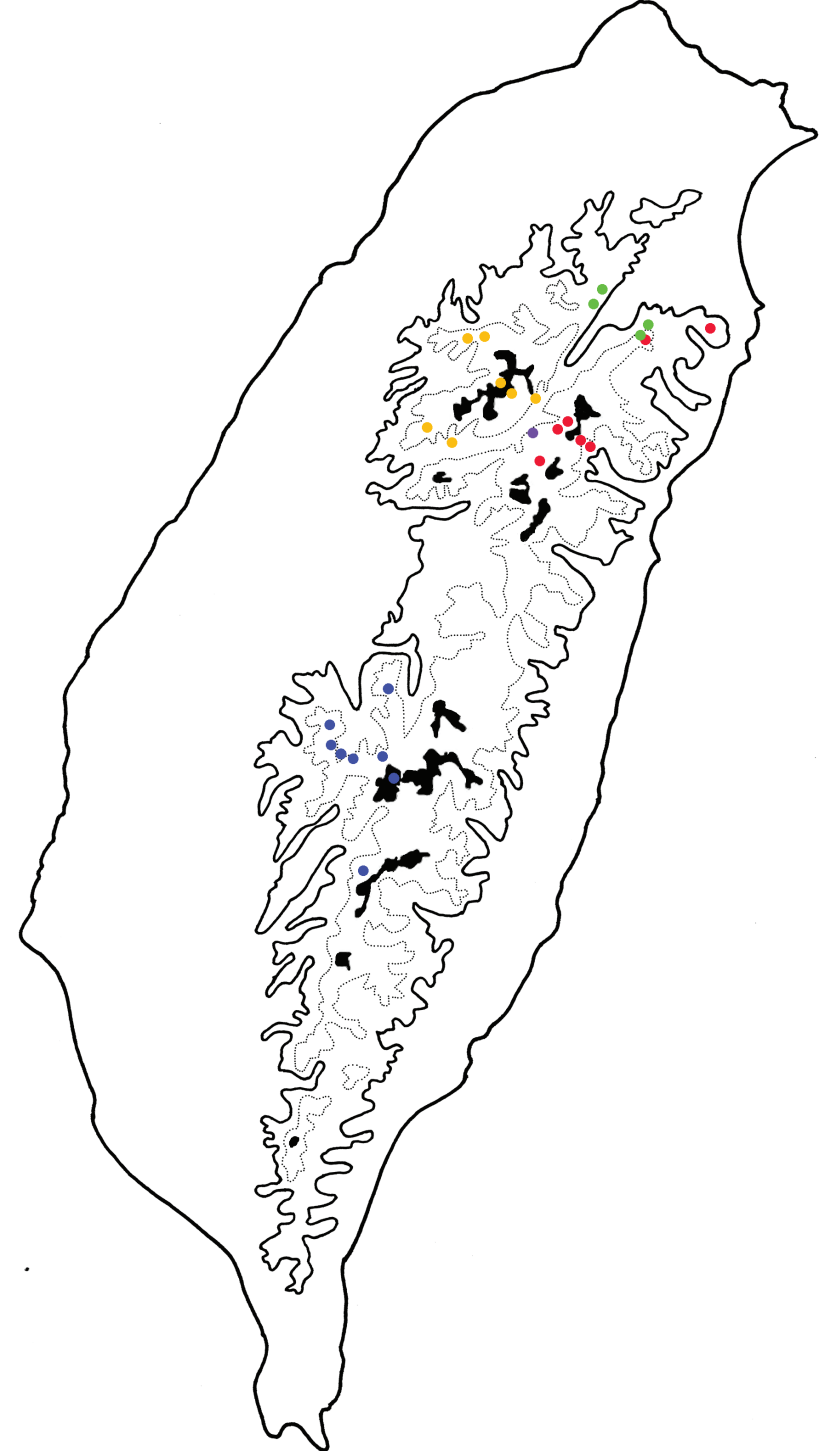
Distribution map of *Furusawaia* species inTaiwan, solid line: 1000 m, broken line: 2000 m, black areas: 3000 m. Key: Red Dots *F.lui* sp. nov. Blue Dots *F.yosonis* Chûjô Green Dots *F.tsoui* sp. nov. Yellow Dots *F.tahsiangi* sp. nov. Purple Dot *F.jungchani* sp. nov.

Only known from the type locality (Fig. 7).

##### Etymology.

The species name is dedicated to Mr Jung-Chan Chen (陳榮章) who collected all specimens of this new species.

#### Furusawaia lui sp. nov.

Taxon classificationAnimaliaColeopteraChrysomelidae

EFAE11EF-E59D-5236-A02E-85BF99759A5E

http://zoobank.org/A03781BE-A4ED-49A4-A7E9-B2D2643C6657

**Figure 8.**
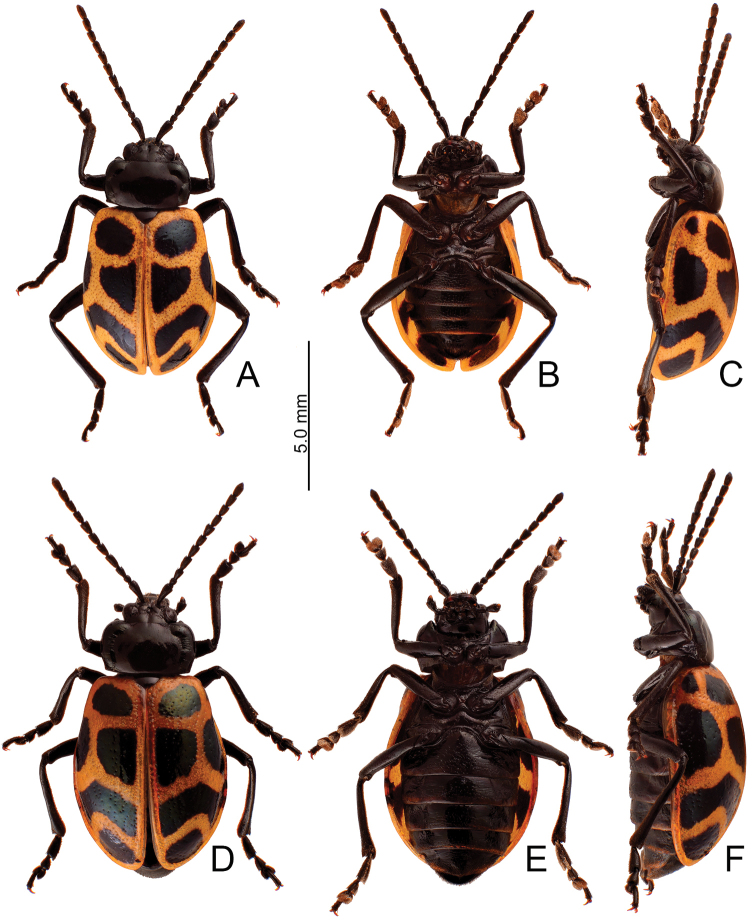
Habitus, *Furusawaialui* sp. nov. **A** male, dorsal view **B** ditto, ventral view **C** ditto, lateral view **D** female, dorsal view **E** ditto, ventral view **F** ditto, lateral view.

**Figure 9.**
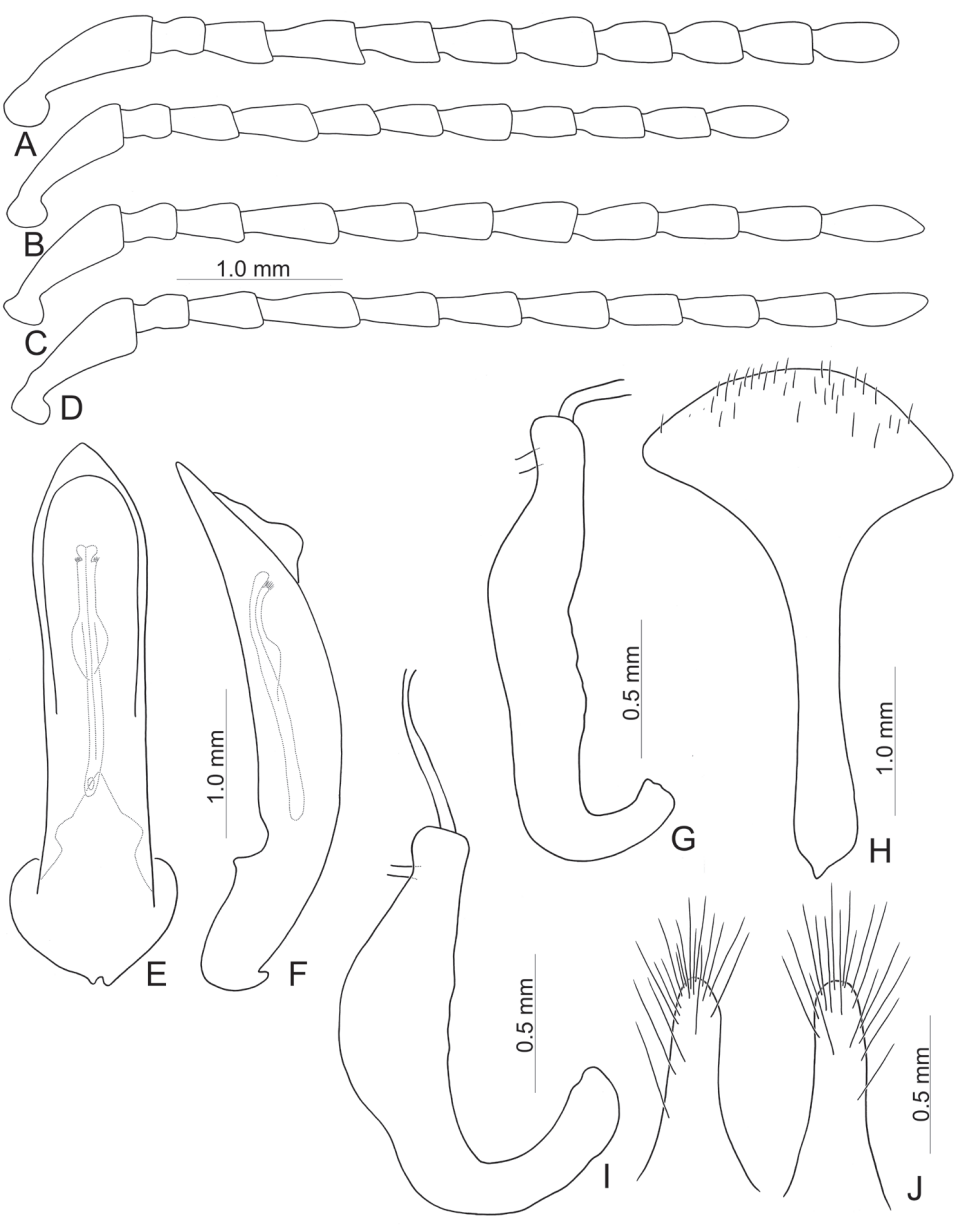
Diagnostic characters of *Furusawaialui* sp. nov. **A** antenna, male, Hsiaofengkou (小風口) **B** antenna, female, Pilu (畢祿) **C** antenna, male, Hsinpaiyang (新白楊) **D** antenna, female, Pilu (碧綠) **E** aedeagus, dorsal view **F** ditto, lateral view **G** spermatheca **H** abdominal ventrite VIII, female **I** spermatheca, variation **J** gonocoxae.

Figs 1A, C, D , 8 , 9

Furusawaia yosonis Chûjô, 1962: 108 (part).

##### Types (n = 21).

***Holotype*** ♂ (TARI), Taiwan. Hualien: Pilu (碧綠), 27.IV.2018, leg. H.-F. Lu (陸錫峯). ***Paratypes***. 1♀ (TARI), same data as holotype; 1♀ (TARI), same but with “25.V.2018”; 1♀ (TARI), same but with “17.VI.2018”; 1♀ (TARI), same but with “23.VI.2018”; Hualien: 1♂, 1♀ (TARI), Hsiaofengkou (小風口), 11.V.2017, leg. C.-T. Yao (姚正得); 1♂ (TARI), same but with “22.VI.2017”; 1♂ (TARI), same locality, 2.VIII.2017, leg. 何彬宏 (B.-H. Ho); 1♂ (TARI), Hsinpaiyang (新白楊), 18.V.2018, leg. H.-F. Lu; 1♂, 2♀♀ (TARI), Karenko (= Hualien, 花蓮), 20.VII.-4.VIII.1919, leg. T. Okuni; Ilan: 1♀ (TARI), Lankanshan (蘭坎山), 1.VII.2017, leg. P.-Y. Chen (陳柏彥); 1♂ (JBCB), Taipingshan (太平山), 19.VI.2008, leg. S.-F. Yu (余素芳); Nantou: 2♀♀ (TARI), 820 Forest road (820 綠林道), 6.VII.2015, leg. T.-H. Lee (李大翔); Taichung: 1♂, 2♀ (TARI), Pilu (畢祿), 7.VII.2015, leg. C.-F. Lee (李奇峯); 1♀ (TARI), same locality, 2.VI.2016, leg. Y.-T. Chung (鍾奕霆).

##### Description.

Length 8.4–9.3 mm, width 4.8–5.5 mm. Body color (Fig. 8) black, elytra with pink (yellow in dead specimens) stripes along basal and lateral margins, and suture, three transverse pink stripes at basal 1/4, middle, and apical 1/4 respectively, anterior stripe curved posteriorly and connected with basal stripe, median angular at middle and connected anteriorly with anterior stripe, posterior stripe angular at middle, basal stripe extending posteriorly from humeral calli and connected with anterior stripe. Antennae filiform in males (Fig. 9A), length ratios of antennomeres I–XI 1.0: 0.3: 0.4: 0.6: 0.5: 0.5: 0.5: 0.5: 0.4: 0.4: 0.5, length to width ratios of antennomeres I–XI 3.3: 1.4: 1.4: 2.1: 1.9: 1.8: 2.0: 1.9: 1.7: 1.7: 2.1; similar in females (Fig. 9B), length ratios of antennomeres I–XI 1.0: 0.3: 0.4: 0.5: 0.4: 0.4: 0.5: 0.4: 0.5: 0.4: 0.5, length to width ratios of antennomeres I–XI 3.2: 1.4: 1.8: 2.3: 2.2: 2.0: 2.1: 2.1: 2.2: 2.2: 2.6. Pronotum 1.8–1.9 × wider than long, disc strongly convex; smooth, without reticulate microsculpture; with punctures obsolete; with lateral impressions; lateral margins distinct including anterior angles, rounded and widest at apical 1/3; apical and basal margin straight; anterior angles strongly produced to a bulbous point. Elytra with rounded lateral margin, widest behind middle, 1.2–1.3 × longer than wide; disc smooth, with sparse, coarse punctures. Aedeagus (Fig. 9E, F) slender in dorsal view, 5.8 × longer than wide, parallel-sided, narrowed near apex, apex narrowly rounded; ostium large, membranous; slightly curved in lateral view; endophallic sclerite elongate, 0.4× as long as aedeagus, widened only at middle, one pair of short lateral expansions near apex, covered with fine setae. Only apices of gonocoxae (Fig. 9J) sclerotized, elongate, apex narrowly rounded, with extremely dense, short setae near apex. Ventrite VIII (Fig. 9G) with apex well sclerotized, several short setae along apical margin, spiculum short. Receptacle of spermatheca (Fig. 12G, I) moderately or slightly swollen, with apex truncate, undivided from pump; pump long and strongly curved; sclerotized proximal spermathecal duct separated from receptacle, short to moderately long.

**Figure 10.**
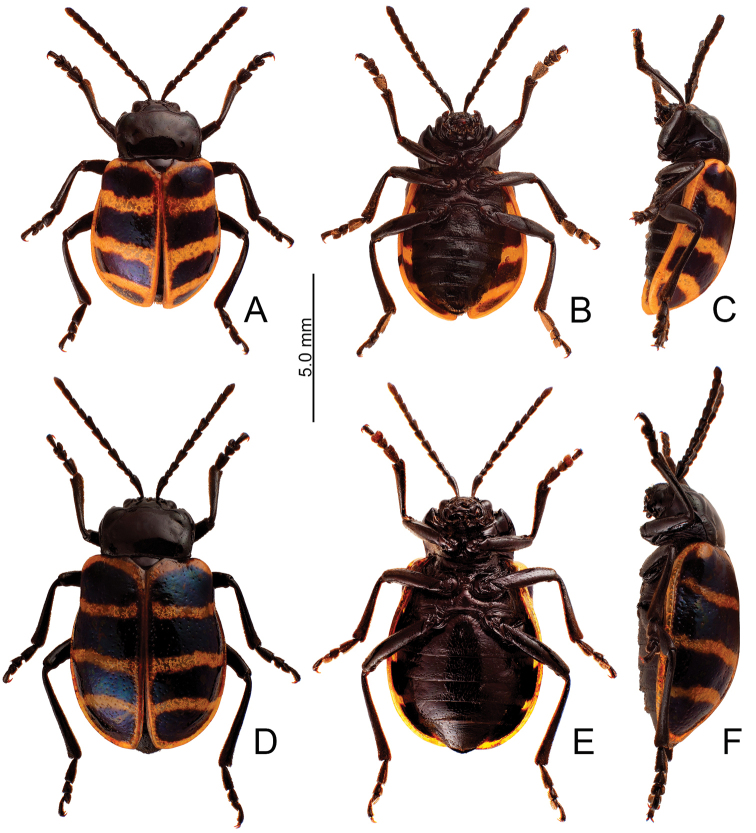
Habitus, *Furusawaiatahsiangi* sp. nov. **A** male, dorsal view **B** ditto, ventral view **C** ditto, lateral view **D** female, dorsal view **E** ditto, ventral view **F** ditto, lateral view.

##### Variations.

Populations in lower altitudes such as Pilu (碧綠, 2200 m) and Hsinpaiyang (新白楊, 1600 m) have more slender antennae, length ratios of antennomeres I–XI in males 1.0: 0.4: 0.5: 0.7: 0.5: 0.5: 0.6: 0.6: 0.6: 0.6: 0.5 (Fig. 9C), length to width ratios of antennomeres I–XI 2.9: 1.5: 1.6: 2.4: 2.1: 2.1: 2.1: 2.3: 2.2: 2.4: 2.9; similar in females (Fig. 9D), length ratios of antennomeres I–XI 1.0: 0.4: 0.5: 0.6: 0.6: 0.5: 0.5: 0.5: 0.5: 0.5: 0.6, length to width ratios of antennomeres I–XI 2.7: 1.6: 1.9: 2.6: 2.5: 2.4: 2.3: 2.4: 2.4: 2.5: 2.9. Northern populations, including specimens collected from Taipingshan and Lankanshan, have less convex pronota.

##### Diagnosis.

**Figure 11.**
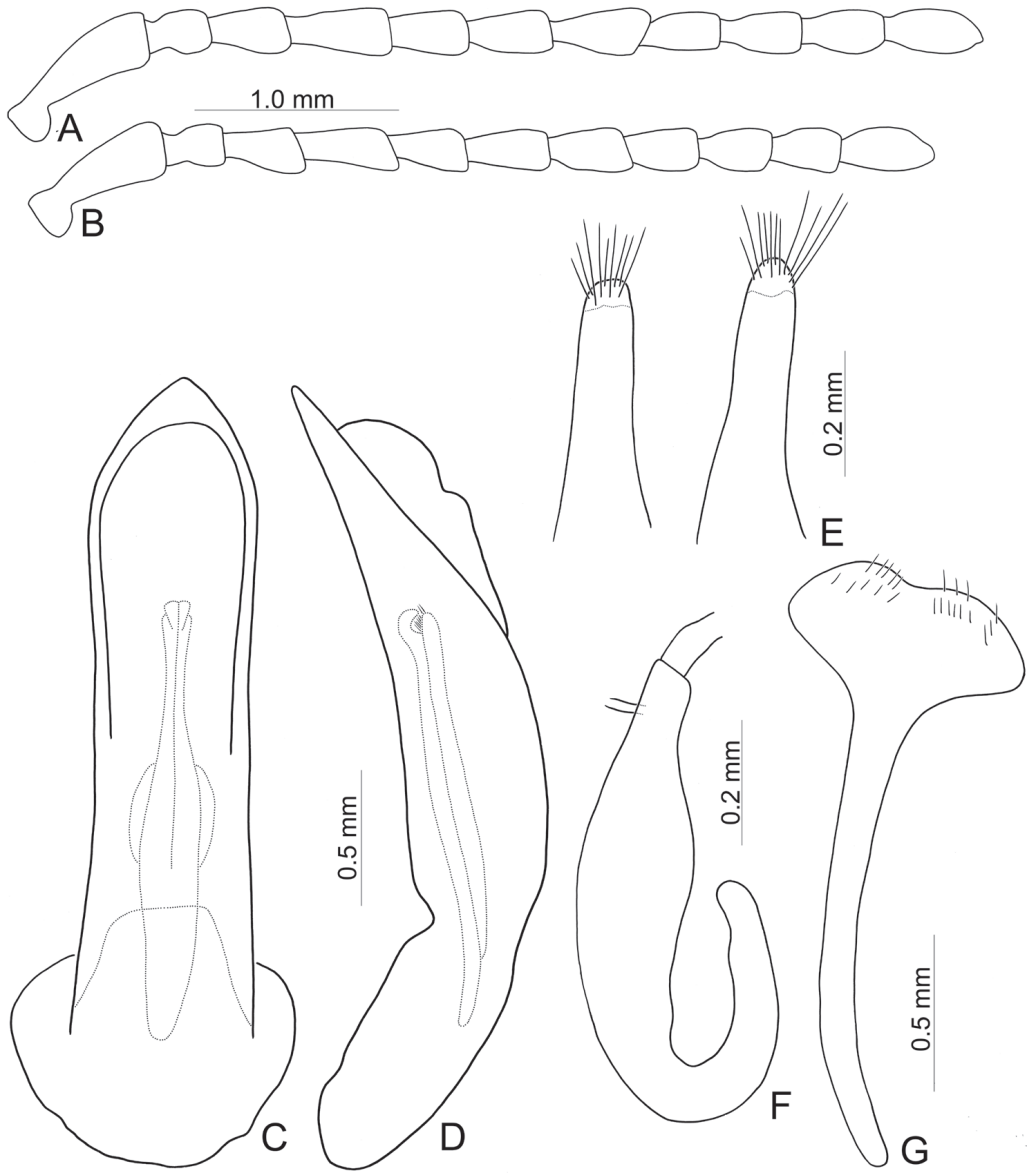
Diagnostic characters of *Furusawaiatahsiangi* sp. nov. **A** antenna, male **B** antenna, female **C** aedeagus, dorsal view **D** ditto, lateral view **E** gonocoxae **F** spermatheca **G** abdominal ventrite VIII, female.

Adults of *Furusawaialui* sp. nov. are characterized by the longitudinal stripes connected between basal and anterior stripes, and anterior and median stripes (Fig. 8). In males of *F.lui* sp. nov., the aedeagus (Fig. 9F) is similar to that of *F.tahsiangi* sp. nov. (Fig. 11D) and *F.tsoui* sp. nov. (Fig. 13D), slightly curved in lateral view (strongly curved in *F.jungchani* sp. nov. (Fig. 6D), moderately curved in *F.yosonis* (Fig. 15D)); endophallic sclerite only widened at middle (Fig. 9E) (basal 2/3 widened and parallel-sided in *F.jungchani* sp. nov. (Fig. 6C) and *F.yosonis* (Fig. 15C), basal 2/3 widened but basally narrowed, and strongly widened at middle in *F.tahsiangi* sp. nov. (Fig. 11C); basal 2/3 widened but basally and at basal 3/7 narrowed in *F.tsoui* sp. nov. (Fig. 13C)). In females of *Furusawaialui* sp. nov., the spermatheca (Fig. 9G, I) is similar to those of *F.tahsiangi* sp. nov. (Fig. 11F), *F.jungchani* sp. nov. (Fig. 6G), and *F.yosonis* (Fig. 15G) with moderately or slightly swollen receptacle (strongly swollen receptacle in *F.tsoui* sp. nov. (Fig. 13G)) and apex truncate and divided from sclerotized proximal duct (apex undivided from sclerotized proximal duct in *F.jungchani* sp. nov. (Fig. 6G), *F.tsoui* sp. nov. (Fig. 13G), and *F.yosonis* (Fig. 15G)); abdominal ventrite VIII (Fig. 9H) is similar to that of *F.jungchani* sp. nov. (Fig. 6F) with well sclerotized and large apex (membranous apex in *F.tsoui* sp. nov. (Fig. 13F) and *F.yosonis* (Fig. 15F); sclerotized and smaller apex in *F.tahsiangi* sp. nov. (Fig. 11G)); the gonocoxae (Fig. 9J) are similar in most Taiwanese species with rounded apices (pointed apices in *F.yosonis* (Fig. 15E)) but dense setae at apical and lateral areas (dense setae only at apical area in *F.jungchani* sp. nov. (Fig. 6E), *F.tahsiangi* sp. nov. (Fig. 11E), and *F.tsoui* sp. nov. (Fig. 13E)).

**Figure 12.**
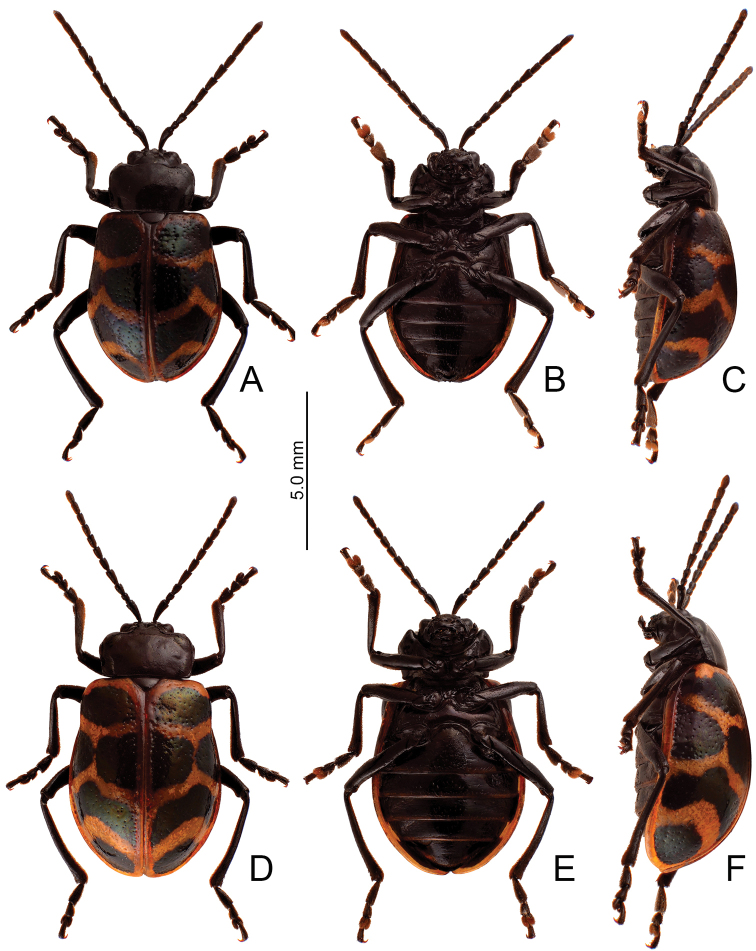
Habitus, *Furusawaiatsoui* sp. nov. **A** male, dorsal view **B** ditto, ventral view **C** ditto, lateral view **D** female, dorsal view **E** ditto, ventral view **F** ditto, lateral view.

**Figure 13.**
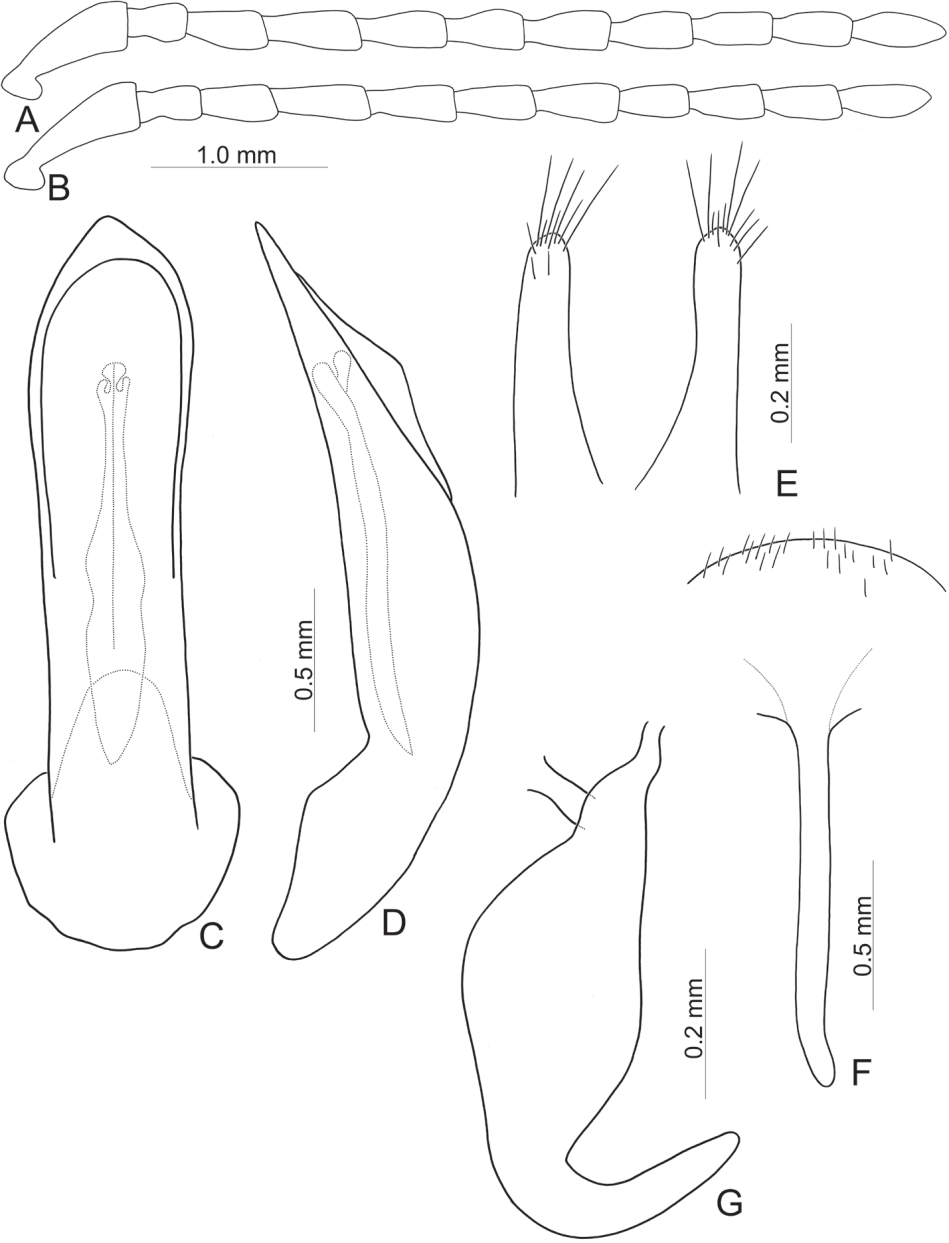
Diagnostic characters of *Furusawaiatsoui* sp. nov. **A** antenna, male **B** antenna, female **C** aedeagus, dorsal view **D** ditto, lateral view **E** gonocoxae **F** abdominal ventrite VIII, female **G** spermatheca.

##### Remarks.

Three paratypes of *Furusawaiayosonis* labeled as “Formosa / Karenko (= Hualien, 花蓮), -19 / VII 20-VIII 4. / T. Okuni [p, w] // Para / Type [p, green letters, circle label with border // Furusawaia / yosonis / CHÛJÔ [h] / DET. M. CHUJO [p, w] // 1912 (♂), 1914 (♀), 2168 (♀) [p, w]” belong to this new species and are designated as paratypes.

##### Food plants.

*Stellariamedia* (L.) Vill (Caryophyllaceae).

##### Biological notes.

Adults were active on forest trails during daytime at Hsinpaiyang (新白楊) (Fig. 3A, C), Pilu (碧綠), and Hsiaofengkou (小風口). They were nocturnal at Pilu (畢祿) (Fig. 3D).

##### Distribution.

This new species is widespread between middle and high altitudes (above 1,500) in north and east Taiwan (Fig. 7). It is sympatric with *F.tsoui* sp. nov. in Taipingshan.

##### Etymology.

The species name is dedicated to Mr Hsi-Feng Lu (陸錫峯), the member of TCRT who collected most specimens of this new species.

#### Furusawaia tahsiangi sp. nov.

Taxon classificationAnimaliaColeopteraChrysomelidae

3A4B4D75-D8E2-5491-8EE9-44280D1C1611

http://zoobank.org/17C8C5CC-7C9C-4EC7-B3AA-7266AD638AFB

Figs 3E , 10 , 11

Furusawaia yosonis Chûjô, 1962: 108 (part).

##### Types (n = 17).

***Holotype*** ♂ (TARI), Taiwan. Taichung: Tahsuehshan (大雪山), 7.VI.2010, leg. T.-H. Lee (李大翔). ***Paratypes*.** 1♀, same data as holotype; Hsinchu: 1♀ (TARI), Kuanwu (觀霧), 4.IV.2017, leg. C.-Y. Chuang (莊靜宜); Ilan: 1♂, 1♀ (TARI), Siyuanyakou (思源啞口), 30.III.2015, leg. P.-H. Ko (柯品薰); Miaoli: 1♂ (TARI), Kueishan (檜山), 27.VII.2020, leg. S.-F. Yu (余素芳); Taichung: 1♂, 1♀ (TARI), Hsuehshan (雪山), 1.IV.2010, leg. W.-B. Yeh (葉文斌); 1♀ (TARI), same locality, 10.VI.2010, leg. P.-L. Tian (田佩玲); 1♂ (TARI), same locality, 1.V.2012, leg. J.-C. Chen (陳榮章); 1♀ (TARI), same locality, 1.V.2012, leg. T.-H. Lee (李大翔); 1♂ (TARI), same locality, 8.V.2015, leg. C.-Y. Hsu (許志遠); 1♂, 1♀ (TARI), same but with “24.IV.2019; 1♀ (TARI), same locality, 20.IV2021, leg. W.-J. Chien (簡畹融); 1♂ (TARI), Hassenzan (= Pahsienshan, 八仙山), 6.VI.1942, leg. A. Mutuura; 1♂ (TARI), Tsuichih (翠池), 1.V.2012, leg. F.-S. Huang (黃福盛).

##### Description.

Length 7.9–8.9 mm, width 4.5–5.5 mm. Body color (Fig. 10) black, elytra with orange stripes along basal and lateral margins, and suture, three transverse orange stripes at basal 1/4, middle, and apical 1/4 respectively, anterior and middle transverse stripes straight, posterior transverse stripe curved slightly anteriorly. Antennae filiform in males (Fig. 11A), length ratios of antennomeres I–XI 1.0: 0.4: 0.5: 0.6: 0.5: 0.5: 0.6: 0.5: 0.5: 0.5: 0.6, length to width ratios of antennomeres I–XI 2.9: 1.5: 1.7: 2.1: 1.8: 1.9: 2.3: 2.1: 2.0: 2.0: 2.3; similar in females (Fig. 11B), length ratios of antennomeres I–XI 1.0: 0.4: 0.5: 0.6: 0.5: 0.5: 0.5: 0.5: 0.5: 0.5: 0.6, length to width ratios of antennomeres I–XI 2.6: 1.5: 1.6: 2.3: 1.8: 2.0: 1.9: 1.7: 1.7: 1.7: 2.2. Pronotum 1.6–1.7 × wider than long, disc slightly convex; smooth, without reticulate microsculpture; with punctures obsolete, with lateral impressions reduced; lateral margins distinct, including anterior angles, rounded and widest at apical 1/3; apical and basal margin straight; anterior angles strongly produced to a bulbous point. Elytra with rounded lateral margin, widest behind middle, 1.2–1.3 × longer than wide; disc smooth, without reticulate microsculpture; and with sparse, coarse punctures. Aedeagus (Fig. 11C, D) slender in dorsal view, 5.5 × longer than wide, parallel-sided, narrowed near apex, apex narrowly rounded; ostium large, membranous; slightly curved in lateral view; endophallic sclerite elongate, 0.5 × as long as aedeagus, basal 2/3 widened but basally narrowed, strongly widened at middle, one pair of short lateral expansions near apex, covered with fine setae. Only apices of gonocoxae (Fig. 11E) sclerotized, elongate, apex narrowly rounded, with dense long setae near apices. Ventrite VIII (Fig. 11G) with apex well sclerotized and small, several short setae along apical margin, spiculum long. Receptacle of spermatheca (Fig. 11F) moderately swollen, undivided from pump, apex truncate; pump long and strongly curved; sclerotized proximal spermathecal duct separated from receptacle, short.

##### Diagnosis.

Adults of *Furusawaiatahsiangi* sp. nov. are similar to those of *F.jungchani* sp. nov. based on the straight anterior and median stripes on the elytra but they differ in the having narrower median and posterior stripes (Fig. 10) (wider median and posterior stripes in *F.jungchani* sp. nov.(Fig. 5)); and less convex pronotum with lateral margin at anterior angles (strongly convex pronotum with reduced lateral margin at anterior angles in *F.jungchani* sp. nov.). In males of *F.tahsiangi* sp. nov., the aedeagus (Fig. 11D) is similar to those of *F.lui* sp. nov. (Fig. 9F) and *F.tsoui* sp. nov. (Fig. 13D), slightly curved in lateral view (strongly curved in *F.jungchani* sp. nov. (Fig. 6D), moderately curved in *F.yosonis* (Fig. 15D)); endophallic sclerite with basal 2/3 wider but basally narrowed, strongly wider at middle (Fig. 11C) (basal 2/3 widened and parallel-sided in *F.jungchani* sp. nov. (Fig. 6C) and *F.yosonis* (Fig. 15C); widened only at middle in *F.lui* sp. nov. (Fig. 9E); basal 2/3 wider, but basally and at basal 3/7 narrowed in *F.tsoui* sp. nov. (Fig. 13C)). In females of *F.tahsiangi* sp. nov., the spermatheca (Fig. 11F) is similar that of *F.lui* sp. nov. (Fig. 9I), with moderately swollen receptacle (slightly swollen receptacle in *F.jungchani* sp. nov. (Fig. 6G) and *F.yosonis* (Fig. 15G); strongly swollen receptacle in *F.tsoui* sp. nov. (Fig. 13G)) and apex truncate and divided from sclerotized proximal duct (apex undivided from sclerotized proximal duct in others); abdominal ventrite VIII (Fig. 11G) well sclerotized, with small apex (membranous apex in *F.tsoui* sp. nov. (Fig. 13F) and *F.yosonis* (Fig. 15F); sclerotized and large apex in *F.jungchani* sp. nov. (Fig. 6F) and *F.lui* sp. nov. (Fig. 9H)); gonocoxae (Fig. 11E) similar to those of *F.jungchani* sp. nov. (Fig. 6E) and *F.tsoui* sp. nov. (Fig. 13E) with rounded apices (pointed apices in *F.yosonis* (Fig. 15E)) and dense setae present only at near apices (dense setae present at apical and lateral areas in *F.lui* sp. nov. (Fig. 9J)).

##### Remarks.

One paratype of *Furusawaiayosonis* labeled: “Taiwan / Hassenzan [p] (= Pahsienshan, 八仙山) / 6.VI.1942 [h] / A. MUTUURA [p, w] // 新八仙山 [h, on the back of the same label] // Para / Type [p, green letters, circle label with border // Furusawaia / yosonis / CHÛJÔ [h] / DET. M. CHUJO [p, w] // 2318 [p, w]”. Chûjô (1962) typed the locality of this specimens as Mt. Shinhassenza which is translated from 新八仙山.

##### Food plants.

*Stellariamedia* (L.) Vill (Caryophyllaceae).

##### Biological notes.

Adults were active on forest trails during daytime from Hsuehshan (雪山) (Fig. 3E) and Siyuanyakou (思源啞口); while they were nocturnal at Tahsuehshan (大雪山).

##### Distribution.

This new species is widespread at high altitudes (above 2,000 m) in central Taiwan (Fig. 7).

##### Etymology.

The species name is dedicated to Mr. Ta-Hsiang Lee (李大翔). He and the first author were the first ones of TCRT to find this new species.

#### Furusawaia tsoui sp. nov.

Taxon classificationAnimaliaColeopteraChrysomelidae

F82DE4ED-6AA6-5932-B5E2-03AE02B0CE72

http://zoobank.org/85943675-C6B4-4E08-A7DF-BFC5B78E7DC4

Figs 3F , 12 , 13

Furusawaia yosonis : Kimoto, 1969: 66 (part).

##### Types (n = 12).

***Holotype*** ♂ (TARI), Taiwan. Ilan: Jianqing trail (見晴步道), 19.V.2015, leg. S.-F. Yu (余素芳). ***Paratypes*.** 1♂, 2♀ (TARI), same data as holotype; Ilan: 1♂ (TARI), Mingchi (明池), 29.VII.2007, leg. M.-H. Tsou (曹美華); 1♀ (BPBM), Taiheizan (= Taipingshan, 太平山); 1♂ (BPBM), same locality, 5.V.1932, leg. J. L. Gressitt; 1♂ (BPBM), same but with “6.VII.1934”; 1♀ (BPBM), same but with “29.VI.193?”; 2♀ (BPBM), same locality, V-VII.1934, leg. L. & M. Gressitt; Hsinchu: 1♀ (TARI), Yuanyanghu (鴛鴦湖), 30.VI.2021, leg. T.-Y. Chien (簡廷仰) & S,-P. Wu (吳書平).

##### Description.

Length 8.1–10.0 mm, width 4.7–5.9 mm. Body color (Fig. 12) black, elytra with pink stripes along basal and lateral margins, and suture, three transverse pink stripes at basal 1/4, middle, and apical 1/4 respectively, anterior stripe subtruncate, median angular at middle, posterior stripe curved upwards, basal stripe extending posterior a little from humeral calli. Antennae filiform in males (Fig. 13A), length ratios of antennomeres I–XI 1.0: 0.4: 0.5: 0.6: 0.6: 0.5: 0.6: 0.6: 0.6: 0.6: 0.7, length to width ratios of antennomeres I–XI 2.9: 1.5: 1.9: 2.4: 2.4: 1.9: 2.2: 2.2: 2.3: 2.3: 2.7; similar in females (Fig. 13B), length ratios of antennomeres I–XI 1.0: 0.4: 0.5: 0.6: 0.6: 0.5: 0.5: 0.4: 0.5: 0.5: 0.6, length to width ratios of antennomeres I–XI 3.2: 1.8: 2.0: 2.7: 2.6: 2.3: 2.2: 2.1: 2.1: 2.3: 2.8. Pronotum 1.7 × wider than long, disc slightly convex; smooth, without reticulate microsculpture; with punctures obsolete, with lateral impressions; lateral margins distinct, rounded, and widest at apical 1/3; apical and basal margin straight; anterior angles strongly produced to bulbous point. Elytra with rounded lateral margin, widest behind middle, 1.2–1.3 × longer than wide; disc smooth, without reticulate microsculpture; and with sparse, coarse punctures. Aedeagus (Fig. 13C, D) slender in dorsal view, 5.8 × longer than wide, parallel-sided, narrowed near apex, apex narrowly rounded; ostium large, covered membranous; slightly curved at apical 1/3 in lateral view; endophallic sclerite elongate, 0.5 × as long as aedeagus, basal 2/3 widened, but basally and at basal 3/7 narrower, one pair of short lateral expansions near apex, covered with fine setae. Only apices of gonocoxae (Fig. 13E) sclerotized, elongate, apex narrowly rounded, with dense, short setae near apices. Ventrite VIII (Fig. 13F) membranous apically, several short setae along apical margin, spiculum long. Receptacle of spermatheca (Fig. 13G) strongly swollen, undivided from pump; pump long and strongly curved; sclerotized proximal spermathecal duct undivided from receptacle, short.

##### Diagnosis.

**Figure 14.**
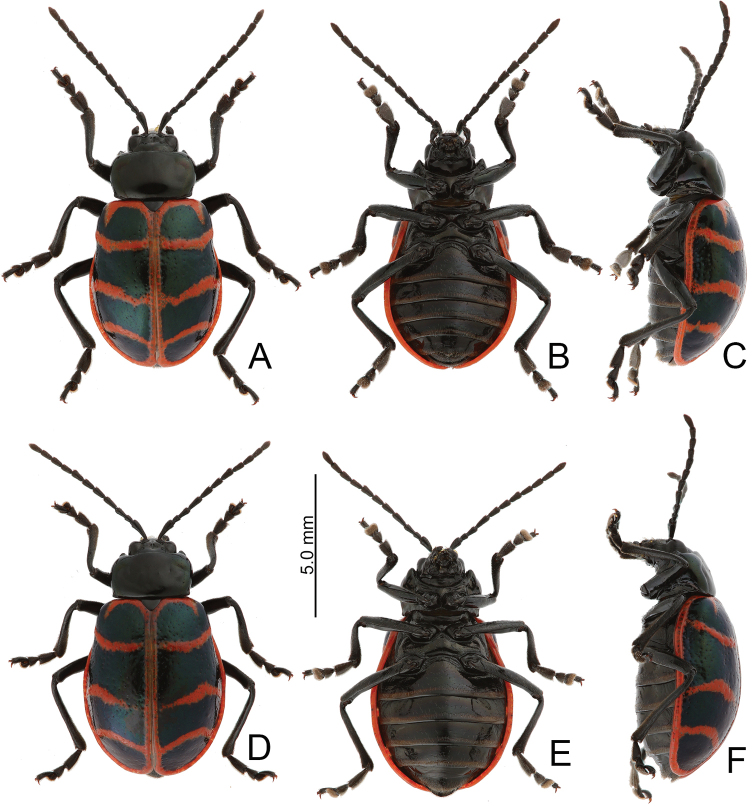
Habitus, *Furusawaiayosonis* Chûjô **A** male, dorsal view **B** ditto, ventral view **C** ditto, lateral view **D** female, dorsal view **E** ditto, ventral view **F** ditto, lateral view.

**Figure 15.**
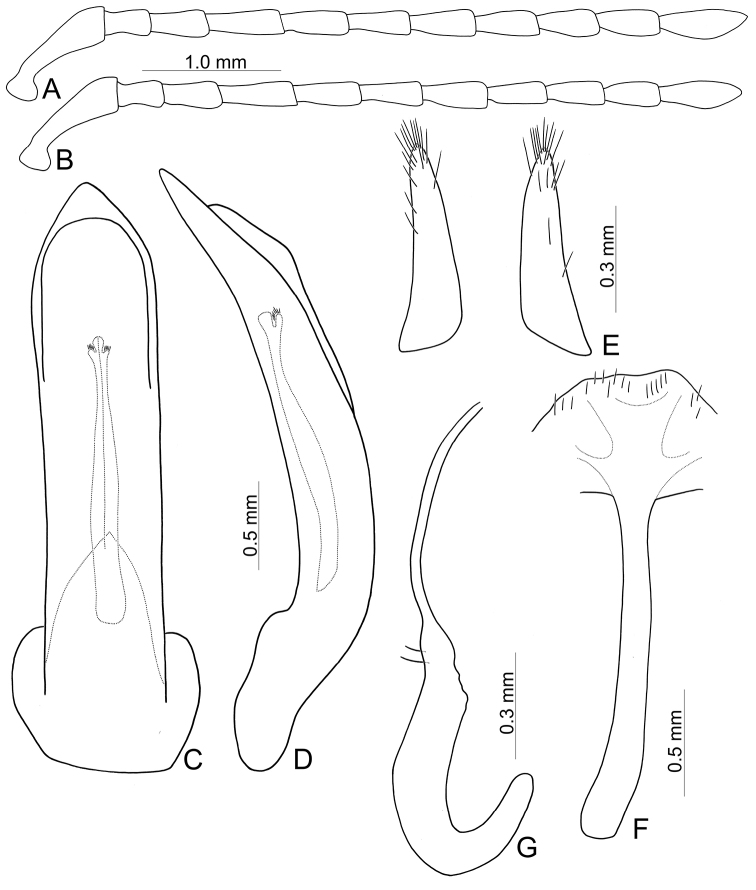
Habitus, *Furusawaiayosonis* Chûjô **A** male, dorsal view **B** ditto, ventral view **C** ditto, lateral view **D** female, dorsal view **E** ditto, ventral view **F** ditto, lateral view.

Adults of *Furusawaiatsoui* sp. nov. (Fig. 12) are similar to *F.yosonis* Chûjô (Fig. 14) based on the curved median and posterior stripes on the elytra (straight median and posterior stripes on the elytra in *F.jungchani* sp. nov. (Fig. 5) and *F.tahsiangi* sp. nov. (Fig. 10)) and without longitudinal stripes connecting basal and anterior stripes, anterior and median stripes (with longitudinal stripes connecting basal and anterior stripes, anterior and median stripes in *F.lui* sp. nov. (Fig. 8)) but differing by the less convex pronotum with lateral margin present at anterior angles (strongly convex pronotum with lateral margin reduced at anterior angles in *F.yosonis*). In males of *F.tsoui* sp. nov., aedeagus (Fig. 13D) similar to those of *F.lui* sp. nov. (Fig. 9F) and *F.tahsiangi* sp. nov. (Fig. 11D), slightly curved in lateral view (strongly curved in *F.jungchani* sp. nov. (Fig. 6D), moderately curved in *F.yosonis* (Fig. 15D)); endophallic sclerite with basal 2/3 widened, but basally and at basal 3/7 narrowed in *F.tsoui* sp. nov. (Fig. 13C) (basal 2/3 widened and parallel-sided in *F.jungchani* sp. nov. (Fig. 6C) and *F.yosonis* (Fig. 15C), widened only at middle in *F.lui* sp. nov. (Fig. 9E), basal 2/3 widened but basally narrowed, strongly widened at middle in *F.tahsiangi* sp. nov. (Fig. 11C)). In females of *F.tsoui* sp. nov., spermatheca (Fig. 13G) with strongly swollen receptacle (slightly or moderately swollen receptacle in others) and apex undivided from sclerotized proximal duct (apex truncate and separate from sclerotized proximal duct in *F.lui* sp. nov. (Fig. 9G, I) and *F.tahsiangi* sp. nov. (Fig. 11F)); abdominal ventrite VIII (Fig. 13F) similar to those of *F.yosonis* (Fig. 15F) membranous apex (well sclerotized small apex in *F.tahsiangi* sp. nov. (Fig. 11G), sclerotized large apex in *F.jungchani* sp. nov. (Fig. 6F) and *F.lui* sp. nov. (Fig. 9H)); gonocoxae (Fig. 13E) similar to *F.jungchani* sp. nov. (Fig. 6E) and *F.tahsiangi* sp. nov. (Fig. 11E) with rounded apices (pointed apices in *F.yosonis* (Fig. 15E)) and dense setae present only near apex (dense setae present at apical and lateral area in *F.lui* sp. nov. (Fig. 9J)).

##### Remarks.

The specimens identified by Kimoto (1969) as *Furusawaiayosonis* collected from Taipingshan (太平山) belong to this new species and are designated as paratypes.

##### Food plants.

*Stellariamedia* (L.) Vill and *Cucubalusbaccifer* L. (Caryophyllaceae).

##### Biological notes.

Adults were active on forest trails during daytime at Mingchi (明池). They were nocturnal on Jianqing trail (見晴步道) (Fig. 3F).

##### Distribution.

This species is widespread at low and mid-altitudes (above 1,000 m) in northern Taiwan (Fig. 7). It is sympatric with *F.lui* sp. nov. in Taipingshan (太平山).

##### Etymology.

The species name is dedicated to Mr Mei-Hua Tsou (曹美華). He was the first to collect adults of this new species in Mingchi (明池).

#### Furusawaia yosonis

Taxon classificationAnimaliaColeopteraChrysomelidae

Chûjô, 1962

9102FA88-1C32-5BF4-8D66-30F4BD369F50

Figs 3G , 14 , 15

Furusawaia yosonis Chûjô, 1962: 109 (Alishan, 阿里山); Kimoto 1969: 66 (part); Wilcox 1971: 210 (catalogue); Kimoto and Chu 1996: 91 (catalogue); Kimoto and Takizawa 1997: 310; Beenen 2010: 458 (catalogue); Yang et al. 2015: 187 (catalogue).

##### Types.

***Holotype*** ♂ (TARI, by original designation): “Holo / Type [p, circle label with letters faded out] // Arisan (= Alishan, 阿里山) / FORMOSA / 24–25.V.1933 / Col. M. CHUJO [p, w] // Furusawaia / yosonis / Chûjô [h] / DET. M. CHUJO [p, w] // 2320 [p, w]”. ***Paratypes***: 1♀ (TARI): “Allo / Type [p, gray letters, circle label with border] // Arisan (= Alishan, 阿里山) / FORMOSA / 24–25.V.1933 / Col. M. CHUJO [p, w] // Furusawaia / yosonis / CHÛJÔ [h] / DET. M. CHUJO [p, w] // 2319 [p, w]”; 1♂ (TARI): “Hunkiko (= Fenchihu, 奮起湖) / VII.6.1928 [p, letters faded out] // R. Takahashi / ???? [p, letters faded, illegible] // Para / Type [p, green letters, circle label with border // Furusawaia / yosonis / CHÛJÔ [h] / DET. M. CHUJO [p, w] // 2319 [p, w]”.

##### Other material (n = 28).

Taiwan. Chiayi: 2♀ (TARI), Arisan (= Alishan, 阿里山), V.1935, leg. Y. Miwa; 1♂ (TARI), same locality, 31.III.1939, leg. A. Aoki; 1♂, 1♀ (TARI), same locality, 20–23.VI.1956, leg. S. C. Chiu; 1♂ (KMNH), same locality, 8.IV.1965, leg. S. Miyamoto; 1♀ (KMNH), 6.VII.1965, leg. T. Yamasaki; 1♂ (TARI), same locality, 17–20.VIII.1982, leg. K. C. Chou & C. C. Pan; 1♂ (JBCB), same locality, 17–26.VI.1995, leg. P. Moravec; 1♀ (TARI), same locality, 26.III.2009, leg. G. Shang (向高世); 1♀ (TARI), same locality, 17.V.2010, leg. T.-H. Lee (李大翔); 1♂, 3♀ (TARI), same locality, 10.V.2021, leg. T.-Y. Chien (簡廷仰); 1♂ (KMNH), Chaoping (沼平), Alishan, 6.VII.1961, leg. S. Ueno; 1♂ (TARI), same locality, 10.V.2021, leg. B.-X. Guo; 1♀ (KMNH), Niitakaguchi (新高山 = Yushankou, 玉山口), – Alishan, 6.IV.1967, leg. T. Shirozu; 1♂ (TARI), Shishan race (石山引水道), 8.III.2020, leg. B.-H. Ho (何彬宏); 1♂ (TARI), Tatachia (塔塔加), 17–24.VII.2008, leg. G.-S. Tung (董景生); 1♂ (TARI), same locality, 7.VI.2009, leg. C.-F. Lee (李奇峯); 1♀ (KMNH), Tzuchung (自忠), 3.VII.1961, leg. S. Ueno; 1♂, 1♀ (TARI), same locality, 8.V.2015, leg. J.-C. Chen (陳榮章); 1♂ (KMNH), Yushan (玉山), 20.V.1981, leg. F. Kimura; Kaohsiung: 1♂ (TARI), Tianchi (天池), 15.IV.2021, leg. F.-S. Huang (黃福盛); Nnatou: 2♀ (KMNH), Tonpoge (= Tungpu, 東埔), 28.III.1967, leg. T. Shirozu.

##### Redescription.

Length 7.8–9.1 mm, width 4.7–6.0 mm. Body color (Fig. 14) black, elytra with red stripes along basal and lateral margins, and suture, three transverse red stripes at basal ¼, middle, and apical ¼ respectively, anterior stripe curved downwards, two posterior stripes curved upwards, basal stripe extending posterior from humeral calli, more or less connected with anterior stripe. Antennae filiform in males (Fig. 15A), length ratios of antennomeres I–XI 1.0: 0.4: 0.4: 0.7: 0.6: 0.5: 0.6: 0.5: 0.5: 0.5: 0.7, length to width ratios of antennomeres I–XI 2.9: 1.8: 1.8: 2.7: 2.8: 2.4: 2.6: 2.3: 2.3: 2.3: 2.9; similar in females (Fig. 15B), length ratios of antennomeres I–XI 1.0: 0.4: 0.5: 0.6: 0.6: 0.5: 0.5: 0.5: 0.5: 0.5: 0.7, length to width ratios of antennomeres I–XI 3.3: 1.7: 2.1: 3.0: 2.7: 2.5: 2.5: 2.6: 2.4: 2.4: 2.9. Pronotum 1.7–1.8 × wider than long, disc strongly convex; smooth, without reticulate microsculpture; with punctures obsolete, with lateral impressions; lateral margins reduced, visible only near basolateral angles, or visible in some individuals, rounded and widest at apical 1/3; apical and basal margin straight; anterior angles strongly produced to bulbous point. Elytra with rounded lateral margins, widest behind middle, 1.2–1.3 × longer than wide; disc smooth, without reticulate microsculpture; and with sparse, coarse punctures. Aedeagus (Fig. 15C, D) slender in dorsal view, 5.9 × longer than wide, parallel-sided, narrowed near apex, apex narrowly rounded; ostium large, membranous; moderately curved in lateral view; endophallic sclerite elongate, 0.4 × as long as aedeagus, basal 2/3 widened and parallel-sided, one pair of short lateral expansions near apex, covered with fine setae. Only apices of gonocoxae (Fig. 15E) sclerotized, elongate, apices pointed, with dense, short setae at near apex, and several short setae along lateral margin. Ventrite VIII (Fig. 15F) membranous apically, several short setae along apical margin, spiculum long. Receptacle of spermatheca (Fig. 15G) as slender as pump, undivided from pump; pump long and strongly curved; sclerotized proximal spermathecal duct undivided from receptacle, extremely long.

##### Diagnosis.

Adults of *Furusawaiayosonis* Chûjô (Fig. 14) are similar to those of *F.tsoui* sp. nov. (Fig. 12) based on the curved median and posterior stripes on the elytra (straight median and posterior stripes on the elytra in *F.jungchani* sp. nov. (Fig. 5) and *F.tahsiangi* sp. nov. (Fig. 10)) and without longitudinal stripes connecting basal and anterior stripes, and anterior and median stripes (with longitudinal stripes connecting basal and anterior stripes, anterior and median stripes in *F.lui* sp. nov. (Fig. 8)). It differs by the more convex pronotum with lateral margins reduced at anterior angles (less convex pronotum with lateral margins present at anterior angles in *F.tsoui* sp. nov.). In males of *F.yosonis*, the aedeagus (Fig. 15D) is moderately curved in lateral view (strongly curved in *F.jungchani* sp. nov. (Fig. 6D), slightly curved in others (Figs 9F, 11D, 13D); the endophallic sclerite (Fig. 15C) similar to that of *F.jungchani* sp. nov. (Fig. 6C) with basal 2/3 wider and parallel-sided (only widened at middle in *F.lui* sp. nov. (Fig. 9E); basal 2/3 widened but basally narrowed, and strongly widened at middle in *F.tahsiangi* sp. nov. (Fig. 11C); basal 2/3 widened, but basally and at basal 3/7 narrower in *F.tsoui* sp. nov. (Fig. 13C)). In females of *F.yosonis* Chûjô, the spermathecae (Fig. 15G) are similar to those of *F.jungchani* sp. nov. (Fig. 6G) with slightly swollen receptacle (moderately swollen receptacle in *F.lui* sp. nov. (Fig. 9I) and *F.tahsiangi* sp. nov. (Fig. 11F); strongly swollen receptacle in *F.tsoui* sp. nov. (Fig. 13G)) and apex undivided from sclerotized proximal duct (apex truncate and separated from sclerotized proximal duct in *F.lui* sp. nov. (Fig. 9G, I) and *F.tahsiangi* sp. nov. (Fig. 11F)); abdominal ventrites VIII (Fig. 15F) are similar to those of *F.tsoui* sp. nov. (Fig. 13F), with membranous apex (well sclerotized and small apex in *F.tahsiangi* sp. nov. (Fig. 11G), sclerotized and larger apex in *F.jungchani* sp. nov. (Fig. 6F) and *F.lui* sp. nov. (Fig. 9H)); gonocoxae (Fig. 15E) with pointed apices (rounded apices in others) and dense setae present only at apical area (dense setae present at apical and lateral area in *F.lui* sp. nov. (Fig. 9J)).

##### Food plants.

*Stellariamedia* (L.) Vill (Caryophyllaceae).

##### Biological notes.

All adults were found on forest trails at night (Fig. 3G).

##### Distribution.

This species is widespread at high altitudes (above 2,000 m) in southern Taiwan (Fig. 7).

### Key to species of *Furusawaia*

**Table:** 

1	Pronotum dull, with reticulate microsculpture, generally flat; elytra with dense, coarse punctures, stripe along suture entirely absent (Fig. 1E, F) or present only from base to basal 1/3 (Fig. 1D).	**2 (Chinese species)**
–	Pronotum shining, without reticulate microsculpture, more or less convex; elytra with sparse coarse punctures, stripes along suture entirely present	**3 (Taiwanese species)**
2	Pronotum with lateral margins rounded, anterior angles obtuse; elytra shining, without reticulate microsculpture, stripe along lateral margin entirely present, stripe along suture only appear from base to basal 1/3 (Fig. 1D)	***F.continentalis* Lopatin**
–	Pronotum with lateral margin narrowed at posterior half, anterior angle strongly produced to bulbous point; elytra dull, with reticulate microsculpture, stripes along lateral margins and suture absent (Fig. 1E, F).	***F.konstantinovi* (Lopatin)**
3	Median and posterior stripes on elytra straight (Figs 5, 10).	**4**
–	Median and posterior stripes on elytra curved (Figs 8, 12, 14).	**5**
4	Pronotum strongly convex, lateral margins reduced behind anterior angles; median and posterior stripes on elytra widened (Fig. 5)	***F.jungchani* sp. nov.**
–	Pronotum less convex, lateral margin visible behind anterior angles; median and posterior stripes on elytra normal (Fig. 10).	***F.tahsiangi* sp. nov.**
5	Basal, anterior, and median stripes on elytra connected by longitudinal stripes (Fig. 8).	***F.lui* sp. nov.**
–	Basal, anterior, and median stripes on elytra separated (Figs 12, 14).	**6**
6	Pronotum strongly convex, lateral margins reduced behind anterior angles.	***F.yosonis* Chûjô**
–	Pronotum less convex, lateral margin visible behind anterior angles.	***F.tsoui* sp. nov.**

## Discussion

Adults of *Furusawaia* Chûjô represent one of the wingless galerucine genera with reduced humeral calli in Taiwan. Most of the wingless galerucines in Taiwan have been studied, including *Taiwanoshaira* Lee & Beenen (2020), *Lochmaea* Weise (Lee 2018), *Shairella* Chûjô (Lee and Beenen 2017), and *Sikkimia* Duvivier (Lee and Bezdĕk 2016). Members of these genera are exclusively nocturnal. However, adults of *Furusawaia* exhibit bizarre behavior in being diurnal or nocturnal in different populations of the same species. Such behaviors may be associated with bicolored elytra, which are unique among wingless galerucines. The function of the bicolored elytra requires further study to determine if they are aposematic or part of a mimicry complex. In addition, the difficulty in collecting adults of *Furusawaia* may result from such bizarre behavior. Adults of wingless galerucines are generally easy to collect by searching food plants at night, except this genus. Usually single or a pair of adults of *Furusawaia* were collected during each field trip based on this collecting method. Citizen scientists play an important role in obtaining sufficient material for study by collecting adults. Twenty citizen scientists were involved and collected about 80% of specimens available for study.

Adults of most *Furusawaia* species are capable for dispersal judging from the distribution maps (Fig. 7), except those of *F.jungchani* sp. nov. Even adults of *F.lui* sp. nov. and *F.tsoui* are sympatric in Taipingshan (太平山). Most adults of the species studied were collected at lower altitudes (belong 2,500 m) where they are easily accessible to collectors. One undescribed female was collected at Tianchi Lodge (天池山莊) (Fig. 3H), 2,860 m, at Nantou county. Some additional undescribed species were found at Mt. Mabolasi (馬博拉斯山, 3,785 m) and Mt. Malichianan (馬利加南山, 3,546 m). They are not described here due to insufficient material. More undescribed species are expected in high mountains that are extremely difficult access. Male aedeagi in the section Capulites are uniform (Bezdĕk and Beenen 2009), and those of Taiwanese *Furusawaisa* species played a minor role in diagnosis. Besides color patterns on the elytra and convexity of pronota, female genitalic characters are more or less diagnostic, including gonocoxae, spermathecae, and abdominal ventrites VIII. Future molecular studies may test the morphological taxonomy and clarify relationships among species.

## Supplementary Material

XML Treatment for Furusawaia

XML Treatment for Furusawaia continentalis

XML Treatment for Furusawaia konstantinovi

XML Treatment for Furusawaia jungchani

XML Treatment for Furusawaia lui

XML Treatment for Furusawaia tahsiangi

XML Treatment for Furusawaia tsoui

XML Treatment for Furusawaia yosonis
